# The effects of Salvia miltiorrhiza and ligustrazine injection combined with ACEI/ARB on diabetic kidney disease: A systematic review and meta-analysis

**DOI:** 10.1097/MD.0000000000035853

**Published:** 2024-02-23

**Authors:** Zixuan Zhang, Lei Luo, Xueling Li, Yifei Zhong

**Affiliations:** a First Branch of Nephrology Department, Longhua Hospital Shanghai University of Traditional Chinese Medicine, Shanghai, China.

**Keywords:** diabetic kidney disease, meta-analysis, salvia miltiorrhiza and ligustrazine injection, systematic review

## Abstract

**Background:**

In China, Salvia miltiorrhiza and ligustrazine (SML) injection are widely used as adjunctive therapy for patients with diabetic kidney disease (DKD). However, different studies have reported conflicting results. Therefore, a systematic review and meta-analysis are necessary to assess the efficacy and safety of SML injection for the treatment of DKD.

**Methods:**

We searched 6 electronic literature databases comparing randomized controlled trials (RCTs) of angiotensin-converting enzyme inhibitor (ACEI)/angiotensin receptor blocker (ARB), SML injection in combination with ACEIs/ARBs that were conducted from inception until September 5, 2023. Two reviewers extracted data and independently assessed the risk of bias. Using the Cochrane Risk of Bias Tool for Risk Assessment. Mean differences (MD) were combined with random-effects models and the corresponding 95% confidence intervals (CI) were reported. Review Manager 5.4 software was used for meta-analysis. Stata 17.0 software was used for sensitivity analysis and Egger test.

**Results:**

The combined results show that the use of SML injection along with ACEI/ARB led to better outcomes than the use of controls in terms of enhancing recovery: renal function: Serum creatinine (MD = −14.69, 95% CI (−19.38, −10.00)), Blood urea nitrogen (MD = −1.23, 95% CI (−1.72, −0.74)), Urinary β2-microglobulin (MD = −4.58, 95% CI (−7.72, −1.44)); urinary protein: Urinary albumin excretion rate (MD = −45.74, 95% CI (−58.92, −32.56)), Urine albumin-creatinine ratio (MD = −11.93, 95% CI (−13.89, −9.96)), 24-h urine proteinuria (MD = −0.59, 95% CI (−0.86, −0.32)), Urine microalbumin (MD = −13.50, 95% CI (−20.18, −6.83)). Additionally, adjuvant therapy with SML injection enhanced results in blood glucose, blood pressure, lipids, and inflammatory responses, and no significant variations in adverse events were discovered between the 2 groups.

**Conclusions:**

In patients with DKD, combining SML injection with ACEI/ARB improves renal function, renal proteinuria, hyperglycemia, blood pressure, dyslipidemia, and inflammatory response.

## 1. Introduction

Diabetes mellitus (DM) is a metabolic disorder caused by insufficient insulin secretion or insulin sensitivity, usually characterized by elevated blood glucose levels,^[1,2]^ and is expected to affect 693 million adults by 2045, compared to the current prevalence of more than 400 million.^[3]^ DM is the leading cause of chronic kidney disease (CKD). Approximately one-third of people with DM, including those with type 1 DM (T1DM) and type 2 DM (T2DM), will develop diabetic kidney disease (DKD).^[4]^ Nearly half of people with DKD will eventually develop end-stage renal disease requiring replacement therapy if not treated properly.^[5,6]^

The basis of DKD treatment includes glycemic control, blood pressure control, and renin-angiotensin-aldosterone system (RAAS) blockade.^[7–9]^ Several trials have shown that angiotensin-converting enzyme inhibitors (ACEIs) and angiotensin receptor blockers (ARBs) reduce the progression of kidney disease and the risk of cardiovascular events.^[10–12]^ RAAS inhibitors have now replaced other drugs as the first-line treatment for patients with DKD.^[13]^ Although ACEIs and ARBs can prolong the lives of patients with CKD, large clinical trials have shown that the combined use of ACEIs and ARBs leads to an increased risk of adverse events such as hyperkalemia and acute kidney injury (AKI),^[14,15]^ which often necessitates the discontinuation of these drugs. Existing treatments delay the onset of DKD, but mortality and morbidity remain high, necessitating broad and comprehensive breakthroughs that promote health and optimize human development across the lifespan,^[16,17]^ into halt the development and progression of DKD.

Salvia miltiorrhiza and ligustrazine (SML) injection is a compound injection composed of the extract from Salvia miltiorrhiza and Ligusticum striatum, has been frequently used for the adjuvant treatment of DKD in China. Clinical studie have found that SML injection can regulate the levels of urine and serum-related factors in DKD patients, improve the reabsorption rate of renal tubules to albumin, and effectively promote the improvement of renal tubule function and renal interstitial fibrosis.^[18,55]^ Some scholars based on the experiment of DKD rat model found that blood urea nitrogen (BUN), 24-h urine proteinuria (24h-UTP) and serum creatinine (SCr) levels in the Salvia miltiorrhiza treatment group were significantly reduced compared with the control group. Combined with the results of kidney histopathological sections and Western blot, the kidney tissue damage in DKD rats accompanied by massive collagen deposition was significantly reduced.^[19]^

Currently, there are more and more articles on the clinical application of SML injection. The number of review studies on its treatment of DKD is also increasing. However these reviews have some limitations, including a 2016 systematic review^[20]^ that reported that SML injection improved the overall efficiency, urinary albumin excretion rate, serum urea nitrogen, serum creatinine, and 24-hour urine protein in patients with DKD. But the article did not mention the effect of SML injection on indicators such as blood glucose, blood lipids, blood pressure and inflammatory factors, and new data from related clinical studies have now been reported. Another systematic review on SML injection for DKD published in 2021^[21]^ included all treatments of conventional Western medicines in the interventions section with a wide range, and did not analyze the subgroups of different Western medicines treatments, and the results of which also have some limitations. Given that RAAS inhibitors are by far the most widely used drugs for the treatment of DKD, and it is unclear whether there is a difference between ACEIs and ARBs in the treatment of DKD in terms of disease regression, the present study summarizes the experience of previous systematic reviews, and comprehensively and systematically collects published clinical studies on the combination of SML injection with ACEIs/ARBs in the treatment of patients with DKD in an attempt to evaluate the efficacy of SML injection in the adjuvant treatment of patients with DKD in a multidimensional and comprehensive manner, to provide more accurate and reliable references for the clinical treatment and the rational use of medication.

## 2. Materials and Methods

### 2.1. Search strategy and study selection

To find relevant papers published up to September 5, 2023, we conducted a series of free systematic literature searches of randomized controlled trials (RCTs) of SML using 6 electronic databases, including PubMed, Web of Science, CNKI, WanFang, VIP, and SinoMed. The following keywords were utilized in the literature search strategy: “Salvia miltiorrhiza and ligustrazine,” “Danshenchuanxiongqin,” “diabetic kidney disease,” “diabetic nephropathy,” “DKD” and “DN.” Synonymous Chinese substitutions were used in the search of Chinese databases. The search strategy for the English databases is shown in Table S1, http://links.lww.com/MD/L671. All potentially eligible studies were evaluated, except for animal studies or reviews. To avoid potentially eligible studies, references were reviewed before their inclusion in the literature. Studies followed PRISMA criteria and were registered with PROSPERO (CRD42023416303).

### 2.2. Eligibility criteria and study selection

Studies were considered for inclusion only if all of the following conditions were met: the original study; the study population was patients diagnosed with T1DM or T2DM with DKD; the study design is RCTs; SML coupled with ACEI/ARB was the intervention method used instead of ACEI/ARB. These medications may be used indefinitely and at any dosage.

Studies that met the following criteria were disqualified: the study did not provide original data (e.g., conference abstract, poster, letter to the editor, supplement, case report, case series, systematic review, and meta-analysis); data could not be extracted from the literature or were incomplete; in duplicate studies of the same study population, only the studies that reported the most complete original data were included.

The eligibility of the included papers was independently determined by 2 researchers (ZZ and LL) after they had read the entire texts, abstracts, and titles of the publications. Disputes were settled by discussion or by a third investigator (YZ).

Main outcome indicators: renal function, urinary protein and adverse reactions. The specific outcomes included: urinary albumin excretion rate (UAER), urinary albumin/creatinine ratio (UACR), SCr, BUN, 24h-UTP, β2-microglobulin (β2-MG), and micro-albumin (mALB).

Secondary outcome indicators: blood glucose, blood pressure, blood lipids, inflammatory markers. The specific outcomes included: 2 hours postprandial blood glucose (2hPBG), fasting plasma glucose (FPG), glycated hemoglobin(HbA1c), systolic blood pressure (SBP), diastolic blood pressure (DBP), triglyceride (TG), cholesterol (TC), high-density lipoproteins cholesterol (HDL-C), low-density lipoprotein cholesterol (LDL-C), interleukin-6 (IL-6), interleukin-18 (IL-18), tumor necrosis factor-α (TNF-α).

### 2.3. Data extraction

Author, title, year, country, design, sample size, register protocol, participant characteristics (diagnostic criteria, age, sex, disease course, baseline levels of outcomes), intervention characteristics (type, dose, duration of intervention, route of administration, length of follow-up), and bias risk information were all taken from each study.

### 2.4. Risk of bias assessment

Using the Cochrane Risk of Bias tool (RoB 1.0), 2 investigators (ZZ and LL) independently evaluated the risk of bias (RoB) of the included studies. For determining bias, RoB 1.0 uses 5 domains: the randomization procedure, departure from planned interventions, missing outcome data, outcome assessment, and choice of the reported outcomes. A research may be categorized into 1 of 3 categories for each domain: high RoB, moderate concerns, or low RoB.

### 2.5. Statistical analyses

In order to choose the best statistical model, we first utilized the χ^2^ test and I^2^ values to determine how heterogeneous the studies were: if *P* ≥ .10 and I^2^ ≤ 50%, there is no statistical difference, we used the fixed effects model; on the contrary, it indicates that the heterogeneity is large, and the random effects model is used for analysis. We next conducted a number of subgroup analyses to look for variables that may cause heterogeneity. The sensitivity analysis was carried out to find out how 1 study might affect the combined findings. The Cochrane Collaboration RevMan 5.3 program was used to analyze the data. Mean difference (MD) was utilized as the treatment effect in continuous variable data, and the confidence interval values were all set to 95%. Based on the length of the intervention (T = 2W, T = 3W, T = 4W, T = 12W, T = 16W), subgroup analyses were conducted. When more than 10 studies were included, Egger test and funnel plots were performed to assess any potential publication bias. The sensitivity analysis was carried out to find out how 1 study might affect the combined findings. Using Stata 17.0, sensitivity analysis and Egger test were carried out.

## 3. Results

### 3.1. Literature search results

**Figure 1.**
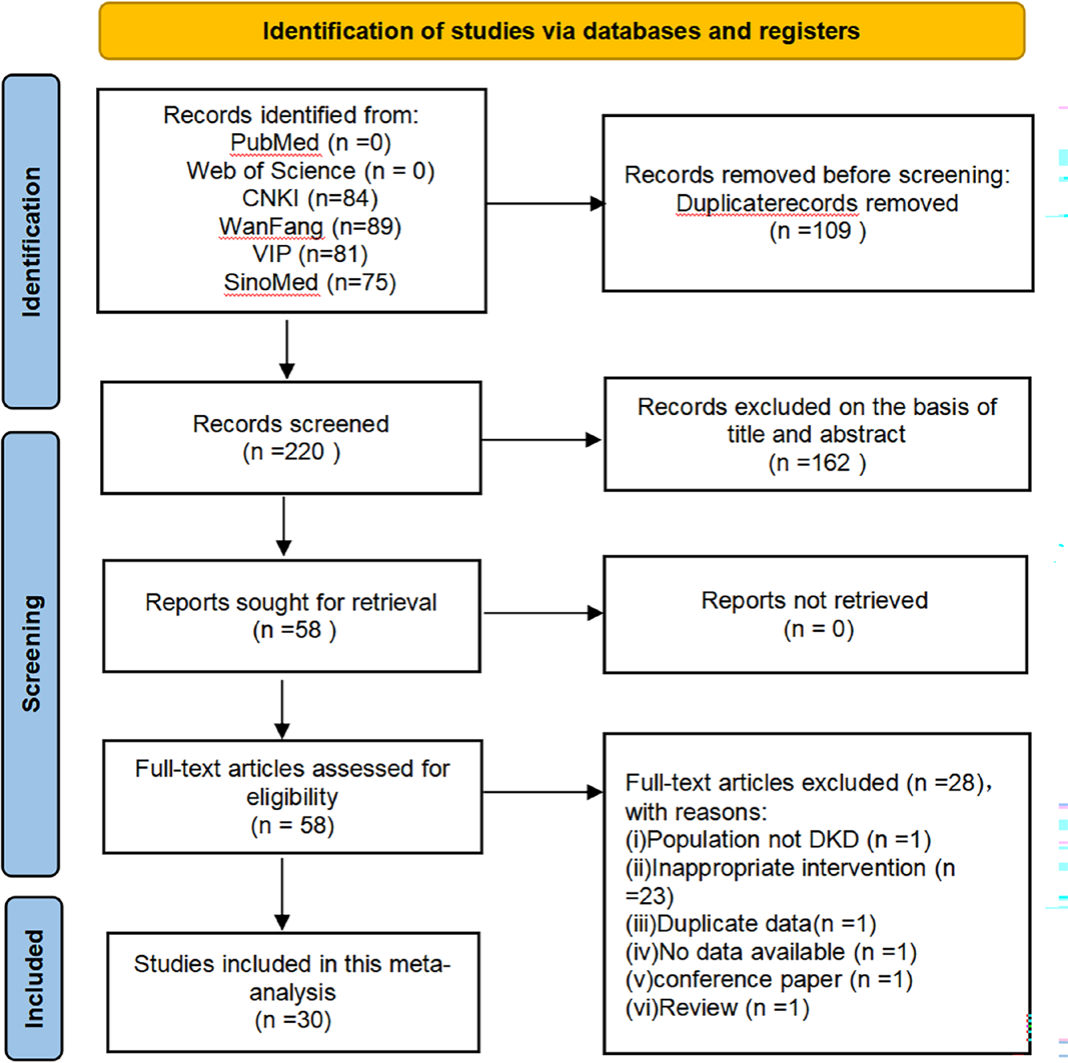
Flow chart of literature search and study selection.

Using the 6 databases mentioned above, we were able to find 329 articles throughout the databases of PubMed (n = 1), Web of Science (n = 1), CNKI (n = 84), WanFang (n = 89), VIP (n = 81) and SinoMed (n = 75). From all records, 110 duplicate articles were removed. We were only reading the titles and abstracts, leaving 163 articles out. 30 studies were finally found suitable for inclusion in the final meta-analysis after carefully studying the entire texts of the 58 included publications. The identification of studies and selection process is illustrated in Figure 1.

### 3.2. Characteristics of included studies

The 30 RCTs included 3214 DKD patients in total (1631 in the experimental group and 1583 in the control group). All of these trials took place on the Chinese mainland between 2011 and 2022, and they were all written in Chinese. The studies that were included had samples sizes ranging from 46 to 360. In this study, the patients’ ages varied from 31 to 64. The length of the treatment ranged from 2 to 16 weeks, with 4 weeks being the norm. There were 17 trials that compared SML combined with ARB to ARB (Zuo, 2018; Fan, 2017; Tao, 2016; Zhang and Li, 2015; Xian, 2015; Zhang, 2014; Yan et al, 2014; Wei et al, 2014; Wu, 2013; Wang and Zhang, 2013; Ke et al, 2013; Li, 2013; Nie, 2012; Zheng et al, 2012; Geng et al Table 1 lists the characteristics of the study.

**Table 1:** Characteristics of studies included in the systematic review and meta-analysis.

Study	Trial period (yr to yr)	Sample size (T/C)	Age/(yr)		Course of disease/(yr)		Gender(Male/Female)		Intervention(s)		Course of treatment	Outcomes	Total efficiency/(%)	
			T	C	T	C	T	C	T	C			T	C
Gong et al, 2022^[22]^	2016.1–2019.12	63/63	55.72 ± 2.69	55.36 ± 2.82	1.81 ± 0.29	1.73 ± 0.36	33/30	34/29	SML 10 mL qd + Fosinopril 20 mg qd	Fosinopril 20 mg qd	2W	24h-UTP, Scr, BUN, 2hPBG, FPG, HbA1c, HDL-C, LDL-C, TC, TG	96.67	79.37
Sun, 2021^[23]^	2017.1–2019.12	60/60	54.10 ± 3.79	54.06 ± 3.45	6.10 ± 1.28	6.07 ± 1.21	39/21	37/23	SML 10 mL qd + Fosinopril 20 mg qd	Fosinopril 20 mg qd	2W	SCr, BUN, LDL-C, HDL-C, TC, TG, 2hPBG, FPG	NA	NA
Tang, 2019^[24]^	2016.9–2017.8	97/83	54.43 ± 5.31	55.02 ± 5.37	6.32 ± 1.21	6.51 ± 1.27	52/45	46/37	SML 10 mL qd + Fosinopril 20 mg qd	Fosinopril 20 mg qd	2W	2hPBG, FPG, HbA1c, TG, 24h-UTP, SCr, BUN, VEGF, ES, IL-6, IL-18, TNF-α	95.88	84.34
Zhuang and Wu, 2019^[25]^	2016.8–2018. 8	194/166	54.1 ± 3.5	55. 4 ± 3. 3	6. 1 ± 1. 3	6. 31 ± 1. 7	104/90	92/74	SML^†^ qd + Fosinopril 20 mg qd	Fosinopril 20 mg qd	2W	24h-UTP, Scr, BUN, 2hPBG, FPG, HbA1c	NA	NA
Zuo, 2018^[26]^	2013.1–2016.1	42/42	31.27 ± 2.64	31.18 ± 2.67	5.14 ± 2.63	5.19 ± 2.67	25/17	26/16	SML 10 mL qd + Irbesartan 150 mg qd	Irbesartan 150 mg qd	2W	UAER, Scr, BUN, HDL-C, TC, TG, DBP, SBP, 2hPBG, FPG	NA	NA
Ma et al, 2018^[27]^	2016.10–2017.9	48/48	46.27 ± 4.11	46.38 ± 4.08	5.52 ± 0.76	5.59 ± 0.75	27/21	29/19	SML 5 ml bid + Benazepril 10 mg bid + Irbesartan 150 mg qd	Benazepril 10 mg bid + Irbesartan 150 mg qd	2W	SCr, BUN, UA, TNF-α, IL-6, IL-8, 2hPBG, FPG, HbA1c	NA	NA
Feng 02018^[28]^	2016.1–2017.12	136/136	59.9 ± 3.43	60.0 ± 3.51	NA	NA	71/65	74/62	SML 5 ml qd + Benazepril 10 mg qd	Benazepril 10 mg qd	2W	UAER, BUN, Scr, Cys C	94.85	79.41
Sun et al, 2017^[29]^	2014.2–2015.9	41/42	52.95 ± 6.83	52.34 ± 6.32	8.67 ± 2.36	8.16 ± 2.47	21/20	21/21	SML 10 ml qd + Benazepril 10 mg bid	Benazepril 10 mg bid	2W	UAER, UACR, β2-MG, IL-6, IL-18, TNF-α	95.12	78.57
Song, 2017^[30]^	2013.9–2016. 12	30/30	57.2 ± 4.7	57.3 ± 4.6	10.3 ± 2.5	10.3 ± 2.4	17/13	18/12	SML 10 ml qd + Benazepril 10 mg bid	Benazepril 10 mg bid	2W	24h-UTP, BUN, 2hPBG, FPG	93.9	70.0
Fan, 2017^[31]^	2013.12–2016.12	75/75	51.86 ± 15.17	52.34 ± 14.65	14.59 ± 6.74	14.36 ± 6.56	43/32	41/34	SML 20 mL qd + Valsartan 80 mg qd	Valsartan 80 mg qd	4W	UAER, Scr, BUN, HbA1c	89.33	66.67
Wang and Chen, 2017^[32]^	2015.3–2017.2	68/68	58.32 ± 7.85	57.72 ± 7.65	10.86 ± 3.69	10.32 ± 3.75	40/28	39/29	SML 200 ml qd + Benazepril 10 mg qd	Benazepril 10 mg qd	4W	UAER, MAP, FPG, ET, mALB, α1-MG, β2-MG	NA	NA
Zhou, 2017^[33]^	2016.2–2017.1	52/52	59.79 ± 2.10	59.28 ± 2.46	5.44 ± 2.46	5.42 ± 2.15	31/21	33/19	SML 40 mg qd + Benazepril 5 mg qd	Benazepril 5 mg qd	4W	Ccr, BUN, 24h-UTP, mALB	96.15	80.77
Tao, 2016^[34]^	2011.3–2016.3	80/80	57.8 ± 4.9	57.2 ± 5.3	10.8 ± 2.4	10.3 ± 2.3	44/36	42/37	SML 10 mL qd + Losartan 50 mg qd	Losartan 50 mg qd	4W	SCr, BUN, DBP, SBP, HbA1c, 2hPBG, FPG	93.75	81.25
Zhang and Li, 2015^[35]^	2014.3–2015.2	23/23	54.12 ± 19.48	54.01 ± 21.23	11.02 ± 5.97	11.31 ± 6.84	13/10	11/12	SML 20 mL qd + Valsartan 80 mg qd	Valsartan 80 mg qd	4W	24h-UTP, BUN, SCr, HbA1c	NA	NA
Xian, 2015^[36]^	NA	38/38	59. 7 ± 10. 5	58. 3 ± 11. 8	8. 7 ± 2. 6	9. 3 ± 3. 2	25/13	24/14	SML 10 mL qd + Valsartan 80 mg qd	Valsartan 80 mg qd	4W	UAER, 2hPBG, FPG, HbA1c, hs-CRP	NA	NA
Tao et al, 2015^[37]^	NA	33/33	53.5 ± 5.8	54.9 ± 6.1	8.7 ± 7.1	8.9 ± 6.8	20/13	18/15	SML 10 ml qd + Benazepril 10 mg bid	Benazepril 10 mg bid	2W	UAER, UACR, β2-MG, IL-6, IL-8, TNF-α	NA	NA
Zhang, 2015^[38]^	2014.3–2015.3	50/50	58.0 ± 8.5	57.0 ± 8.5	11 ± 3	12 ± 2	24/26	28/22	SML 10 mL qd + Irbesartan 150 mg qd	Irbesartan 300 mg qd	2W	UAER, Scr, BUN, HDL-C, TC, TG, DBP, SBP, 2hPBG, FPG	NA	NA
Pan, 2015^[39]^	2012.1–2014.12	36/36	59. 3 ± 5. 4		NA	NA	34/38		SML 10 ml qd + Benazepril 10 mg qd/bid + Fluvastatin 20 mg qd	Benazepril 10 mg qd/bid + Fluvastatin 20 mg qd	4W	24h-UTP, SCr, Hcy, ALB	NA	NA
Zhang, 2014^[40]^	2012.7–2014.1	40/40	60.53 ± 17.45	62.13 ± 18.17	NA	NA	16/24	18/22	SML 10 mL qd + Losartan 50 mg qd	Losartan 50 mg qd	4W	24h-UTP, Scr, BUN, HbA1c, β2-MG	NA	NA
Yan et al, 2014^[41]^	2008.6–2014.1	50/50	31.32 ± 2.51	31.36 ± 2.54	5.2 ± 2.5	5.3 ± 2.4	28/22	28/22	SML 10 mL qd + Irbesartan 150 mg qd	Irbesartan 150 mg qd	12W	Scr, BUN, 2hPBG, FPG, DBP, SBP, mALB	90.0	66.0
Wei et al, 2014^[42]^	2011.3–2013.3	30/30	NA	NA	NA	NA	NA	NA	SML 120 mg qd + Telmisartan 80 mg qd	Telmisartan 80 mg qd	16W	UAER, DBP, SBP, Scr, ALB, BUN, UA, LDL-C, HDL-C, HbA1c	93.3	73.3
Wu, 2014^[43]^	2012.3–2014.3	40/40	55.6 ± 8.4	56.8 ± 9.2	8.2 ± 1.6	6.8 ± 1.2	23/17	22/18	SML 10 ml qd + Irbesartan 150 mg qd	Irbesartan 150 mg qd	12W	UAER, DBP, SBP, Scr, HDL-C, LDL-C, TC, TG	92.5	70.0
Wang and Zhang, 2013^[44]^	2010.9–2012.8	28/23	NA	NA	NA	NA	NA	NA	SML 15–20 mL qd + Valsartan 80 mg qd	Valsartan 80 mg qd	3W	UAER, 2hPBG, FPG, HbA1c, Scr, BUN	NA	NA
Ke et al, 2013^[45]^	NA	32/32	54.63 ± 8.76	55.56 ± 8.21	NA	NA	19/13	17/15	SML 20 mL qd + Valsartan 80 mg qd	Valsartan 80 mg qd	4W	UAER, FPG, HbA1c	NA	NA
Lan, 2013^[46]^	2008.1–2012.10	48/46	57.2 ± 6.9	56.8 ± 7.1	8.9 ± 6.8	9.1 ± 6.9	29/19	27/19	SML 10 ml qd + Benazepril 10 mg bid	Benazepril 10 mg bid	2W	UAER, β2-MG, UACR, IL-6, IL-18, TNF-α	NA	NA
Li, 2013^[47]^	2009.4–2012.1	39/39	32~78		6~28		46/32		SML 10 mL qd + Valsartan 80 mg qd	Valsartan 80 mg qd	4W	24h-UTP, HbA1c, DBP, SBP	NA	NA
Nie, 2012^[48]^	2009.2–2011.2	45/45	58.4 ± 6.3		5~21		48/42		SML 10 mL qd + Losartan 50 mg qd	Losartan 50 mg qd	4W	SCr, BUN, DBP, SBP, 2hPBG, FPG, HbA1c	NA	NA
Zheng et al, 2012^[49]^	2008.6–2011.1	39/39	42 ± 12.2	40 ± 12.6	6~21	5~22	21/18	23/16	SML 10 mL qd + Valsartan 80 mg qd	Valsartan 80 mg qd	4W	24h-UTP, HbA1c, DBP, SBP	NA	NA
Geng et al, 2012^[50]^	2010.12–2012.1	38/38	56.7 ± 10.8	57.2 ± 9.7	12.9 ± 6.9	8.6 ± 5.7	20/18	20/18	SML 10 mL qd + Irbesartan 150 mg qd	Irbesartan 150 mg qd	2W	UAER, BUN, Scr, TG, TC, HDL-C, LDL-C, DBP, SBP, ALT, AST, ALB, GLB	89.47	65.79
Chen, 2011^[51]^	2007–2009	36/36	64. 6 ± 5. 8	63. 4 ± 5. 2	8.2 ± 3.5	8.1 ± 3.4	20/16	19/17	SML 10 mL qd + Valsartan 80~160 mg qd	Valsartan 80~160 mg qd	3W	Scr, BUN, 2hPBG, FPG, HbA1c, DBP, SBP	NA	NA

24h-UTP = 24-h urine proteinuria, 2hPBG = 2 hs postprandial blood glucose, ALB = serum albumin, ALT = alanine aminotransferase, AST = aspartate aminotransferase, BUN = blood urea nitrogen, C = control group, CysC = cystatin C, DBP = diastolic blood pressure, ES = endostatin, ET = endothelin, F = female, FPG = fasting blood glucose, GLB = globulin, HbA1c = glycated hemoglobin, Hcy = homocysteine, IL-18 = interleukin 18, IL-6 = interleukin 6, IL-8 = interleukin 8, M = male, mALB = urine microalbumin, MAP = mean arterial pressure, SBP = systolic blood pressure, Scr = serum creatinine, SML = salvia miltiorrhiza and ligustrazine, T = intervention group, TC = total cholesterol, TG = triglyceride, TNF-α = tumor necrosis factor α, UACR = urine albumin/creatinine ratio, UAER = urine albumin excretion rate, VEGF = vascular endothelial growth facto, α1-MG = urine α1 microglobulin, β2-MG = urine β2 microglobulin.

### 3.3. Assessment of risk of bias

**Figure 2.**
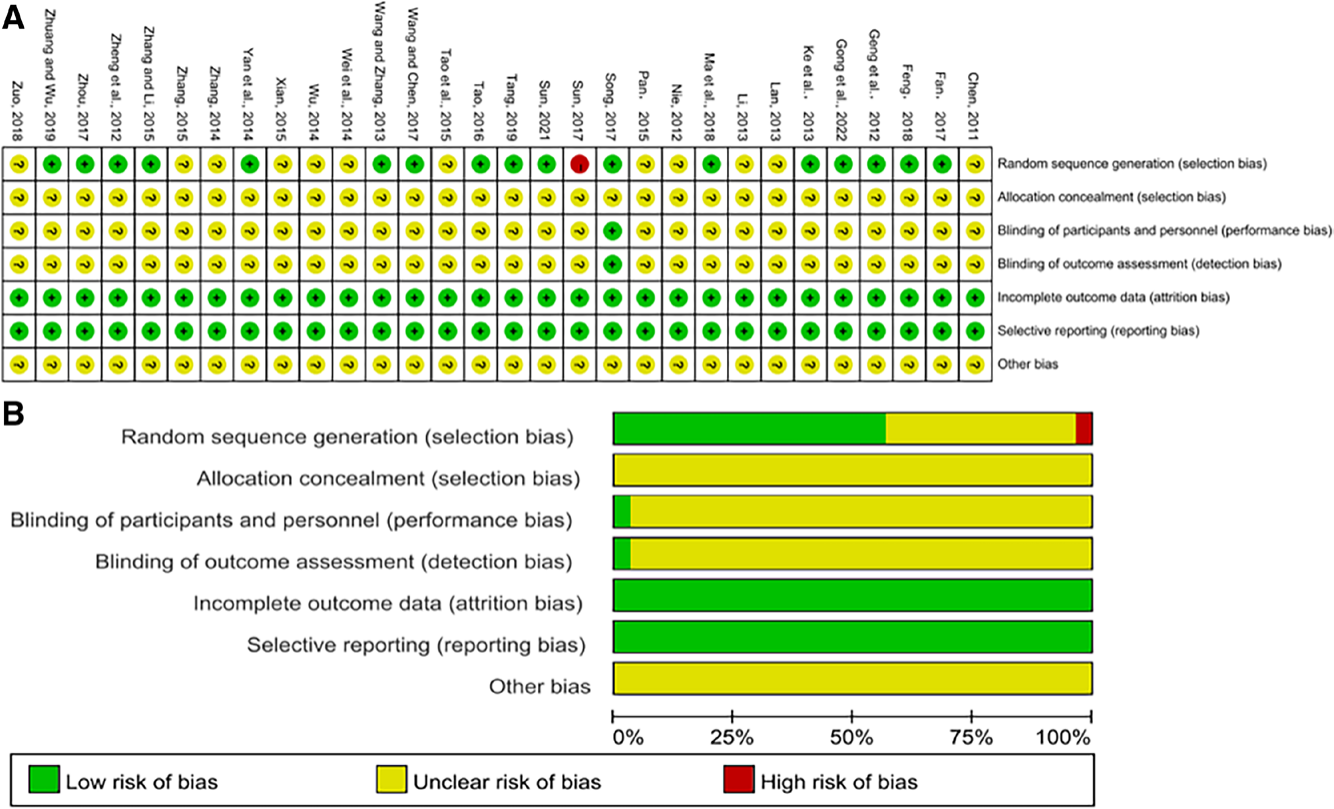
(A) Risk of bias summary about each risk of bias item for each included study. (B) Risk of bias graph about each risk of bias item presented as percentages across all included studies.

The Cochrane Collaboration techniques for assessing bias risk were employed to assess the caliber of the literature. All 30 pieces of literature that were considered for the research had full data and no follow-up reports are missing or lost. 8 research (Gong et al, 2022; Sun, 2021; Tang, 2019; Zhuang and Wu, 2019; Feng, 2018; Zhou, 2017; Yan et al, 2014; Ke et al, 2013; Wang and Zhang, 2013; Geng et al, 2012) implemented randomization using a random number table. One study used the lottery method (Tao, 2016), 1 study used the envelope method (Fan, 2017), and the remaining studies only mentioned randomness without proposing a specific randomizing method. One study mentioned blinding (Song, 2017). Three studies used the odd-even number method to group (Ma et al, 2018; Wang and Chen, 2017; Zhang and Li, 2015). One study used the lottery method. Allocation concealment was not reported in any of the trials. All of the studies provided the intended outcome indicators, including those that were previously described in the published literature, however none of them disclosed the study design; There are no known bias evaluation hazards. As a result, we came to the conclusion that the risk bias of the literature used in this research was “uncertain.” Figure 2 displays the findings of the examination of the risk of bias.

### 3.4. Effect of SML combined with ACEI/ARB against DKD

#### 3.4.1. Effects on renal function.

##### 3.4.1.1. Scr.

**Figure 3.**
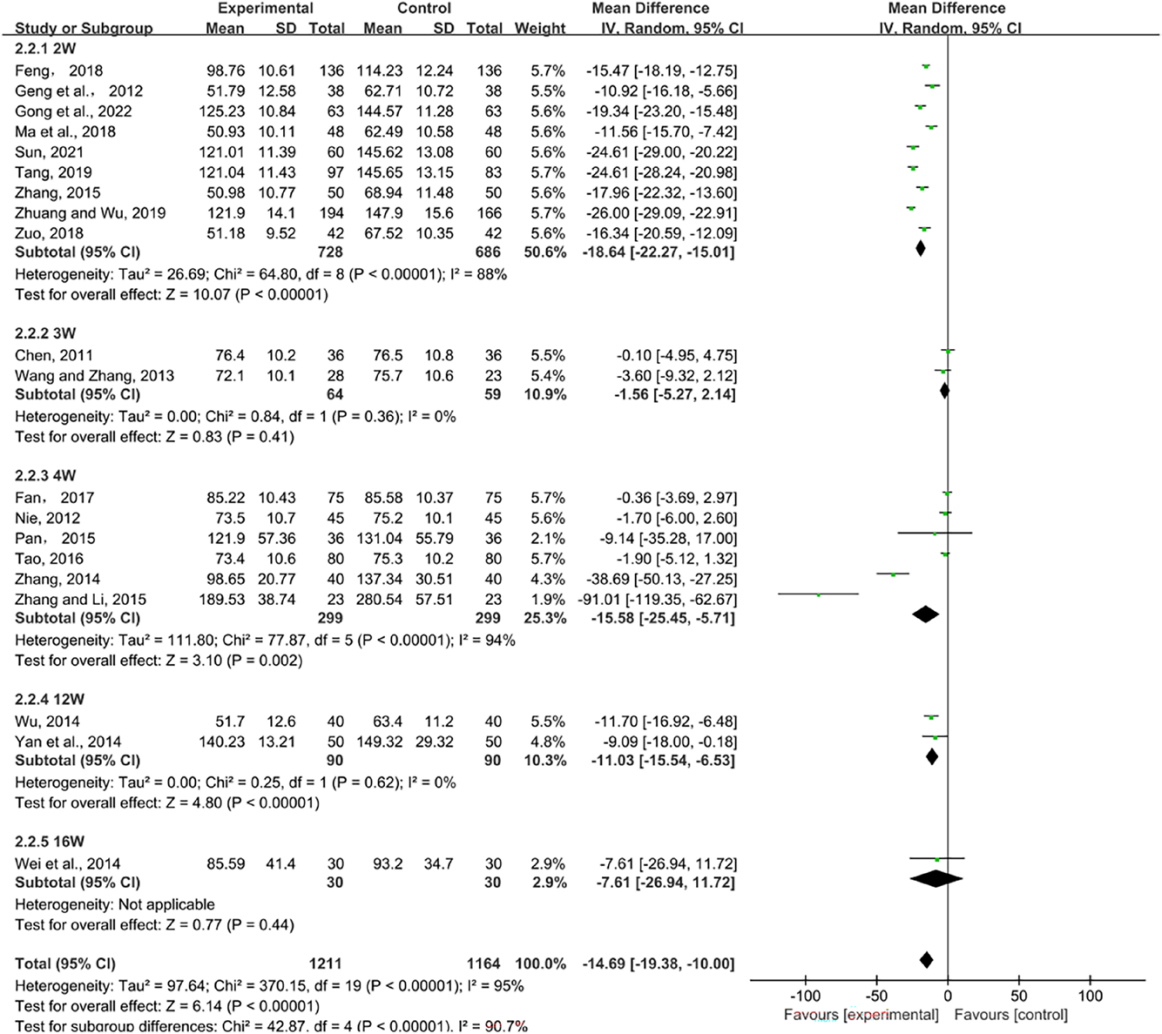
Effects of SML combined with ACEI/ARB vs ACEI/ARB on Scr. SML = salvia miltiorrhiza and ligustrazine.

Twenty RCTs with 2375 individuals looked into the effects of scr. Pooled data were analyzed for significant heterogeneous distribution (*P* < .00001, I^2^ = 95%) using random-effects models (MD = −14.69, 95% CI (-19.38, −10.00). The results demonstrated that compared with the control group of conventional western medicine alone, SML combined with ACEI/ARB could significantly reduce SCr. We divided them into 5 subgroups according to the duration of treatment, and the results of subgroup analysis showed that The combination of 2-week, 4-week and 12-week courses was superior to the control group in reducing SCr, with the 2-week treatment having the best effect (2W: MD = −18.64, 95% CI (−22.27, −15.01), *P* < .00001; 3W: MD = −1.56, 95% CI (−5.27, 2.14), *P* = .41; 4W: MD = −15.58, 95% CI (−25.45, −5.71), *P* = .002; 12W: MD = −11.03, 95% CI (−15.54, −6.53), *P* < .00001; 16W: MD = −7.61, 95% CI (−26.94, 11.72), *P* = .44) (Fig. 3).

##### 3.4.1.2. BUN.

**Figure 4.**
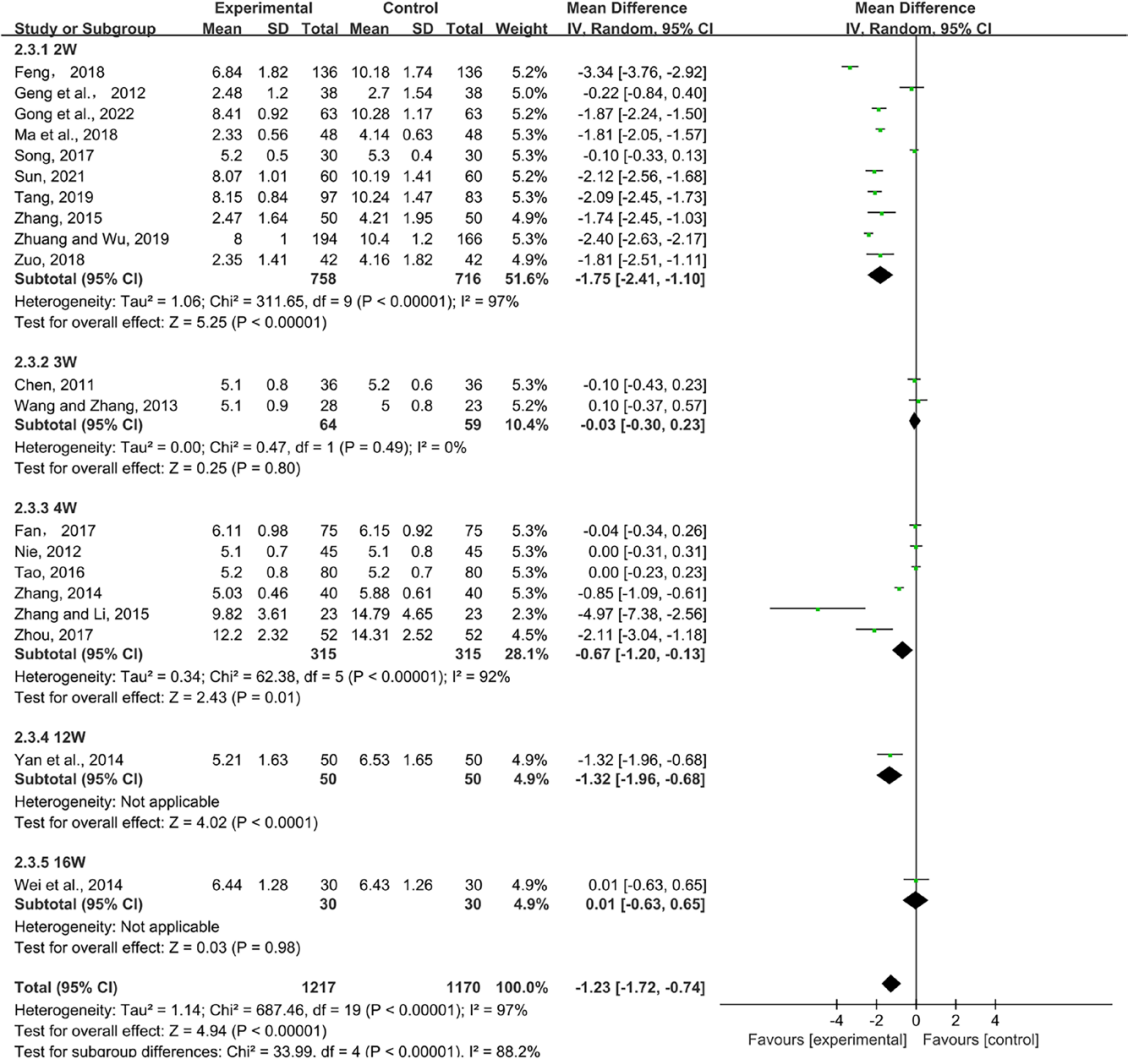
Effects of SML combined with ACEI/ARB vs ACEI/ARB on BUN. SML = salvia miltiorrhiza and ligustrazine.

Pooled data were analyzed for significant heterogeneous distribution (*P* < .00001, I^2^ = 97%) using random-effects models (n = 2387, MD = −1.23, 95% CI (−1.72, −0.74), *P *< .00001). Based on meta-analysis, SML combined with ACEI/ARB reduced BUN significantly compared to ctrl. The 2-week, 4-week and 12-week regimens of the combination were superior to the control group in terms of BUN reduction, respectively (2W: MD = −1.75, 95% CI (−2.41, −1.10), *P* < .00001; 3W: MD = −0.03, 95% CI (−0.30, 0.23), *P* = .80; 4W: MD = −0.67, 95% CI (−1.20, −0.13), *P = *.01; 12W: MD = −1.32, 95% CI (−1.96, −0.68), *P* < .00001; 16W: MD = 0.01, 95% CI (−0.63, 0.65), *P* = .98) (Fig. 4).

##### 3.4.1.3. β2-MG.

**Figure 5.**
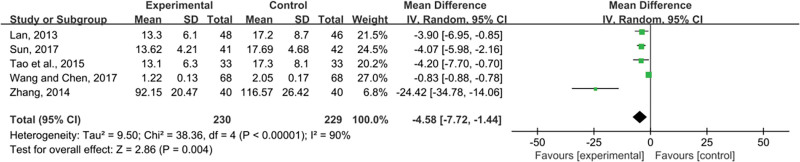
Effects of SML combined with ACEI/ARB vs ACEI/ARB on β2-MG. SML = salvia miltiorrhiza and ligustrazine.

Five studies reported 2-MG. Pooled data (n = 459, MD = −4.58, 95% CI (−7.72, −1.44), *P* = .004) were analyzed for significant heterogeneous distribution (*P* < .00001, I^2^ = 90%) using random-effects models. The intervention group lowered 2-MG more than the control group, according to a meta-analysis (Fig. 5).

#### 3.4.2. Effects on urinary protein.

##### 3.4.2.1. UAER.

**Figure 6.**
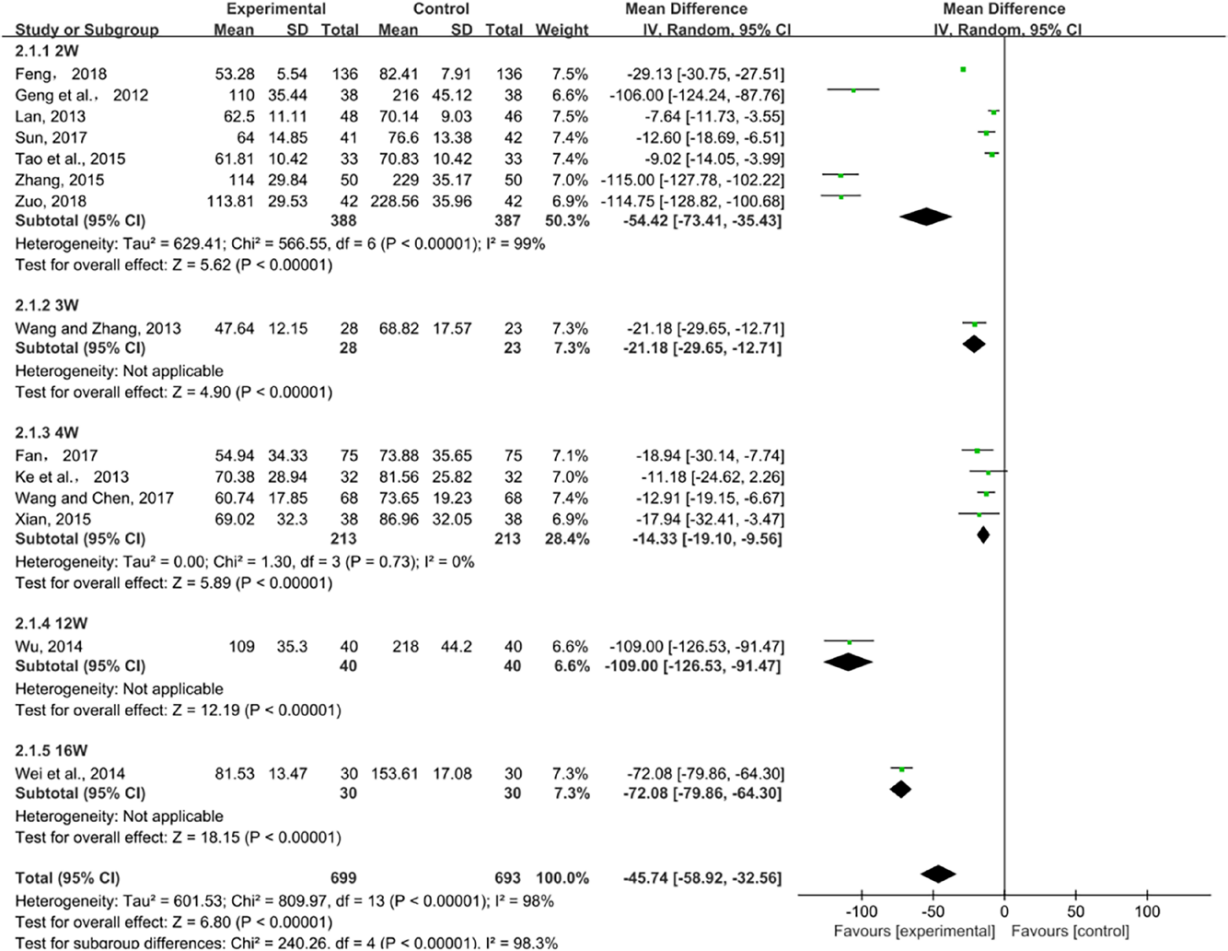
Effects of SML combined with ACEI/ARB vs ACEI/ARB on UAER. SML = salvia miltiorrhiza and ligustrazine.

UAER was reported in 14 studies. Random-effects models were used to analyze pooled data (n = 1392, MD = −45.74, 95% CI (−58.92, −32.56), *P* < .00001) for significant heterogeneous distribution (*P* < .00001, I^2^ = 98%). Based on meta-analysis, the combined intervention reduced UAER more effectively than ACEI/ARB. The results of the intervention time subgroup analysis showed that the combined medication group of different treatment courses was better than the control group in reducing UAER respectively (2W: MD = −54.42, 95% CI (−73.41, −35.43), *P* < .00001; 3W: MD = −21.18, 95% CI (−29.65, −12.71), *P* < .00001; 4W: MD = −14.33, 95% CI (−19.10, −9.56), *P* < .00001; 12W: MD = −109.00, 95% CI (−126.53, −91.47), *P* < .00001; 16W: MD = −72.08, 95% CI (−79.86, −64.30), *P* < .00001) (Fig. 6).

##### 3.4.2.2. UACR.

**Figure 7.**
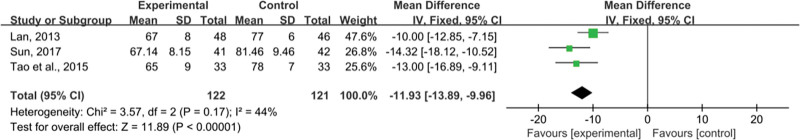
Effects of SML combined with ACEI/ARB vs ACEI/ARB on UACR. SML = salvia miltiorrhiza and ligustrazine.

There was UACR in 3 studies. Pooled data were analyzed using fixed-effects models (n = 243, MD = −11.93, 95% CI (−13.89, −9.96), *P* < .00001). No discernible heterogeneous distribution was found (*P* = .17, I^2^ = 44%) after analysis of the homogeneity statistic. According to the results of the meta-analysis, the intervention group lowered UACR more than the control group (Fig. 7).

##### 3.4.2.3. 24h-UTP.

**Figure 8.**
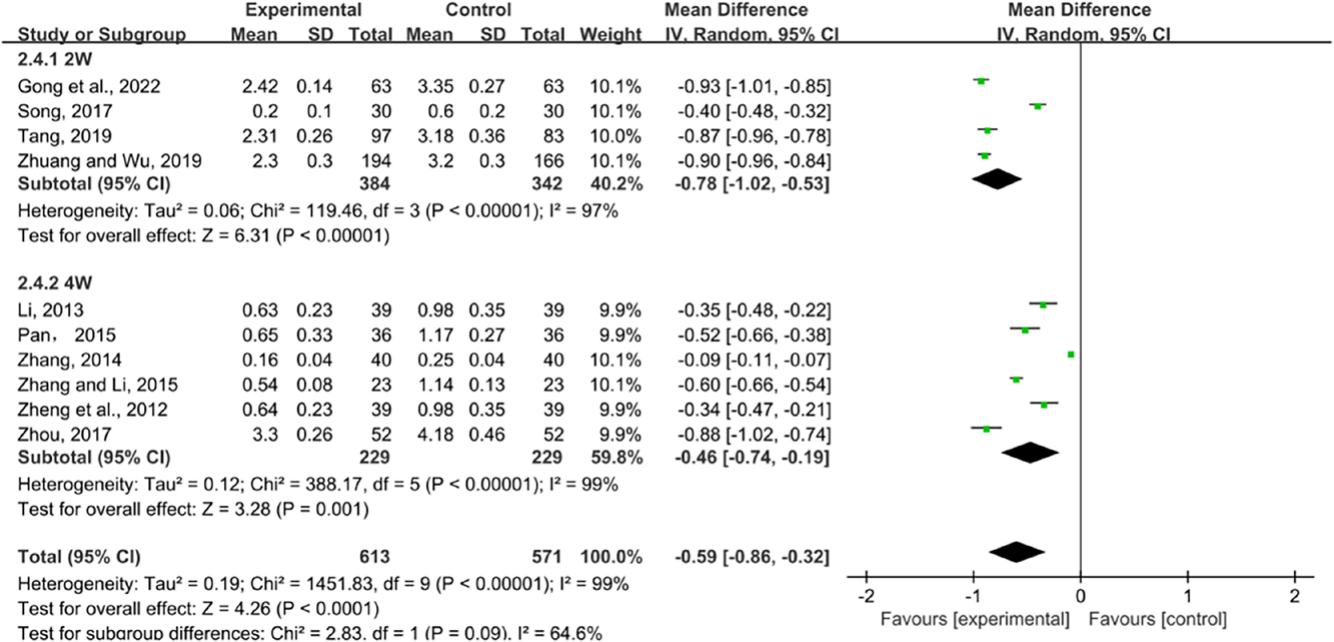
Effects of SML combined with ACEI/ARB vs ACEI/ARB on 24h-UTP. SML = salvia miltiorrhiza and ligustrazine.

Ten studies mentioned 24h-UTP. Random-effects models were used to analyze pooled data (n = 1184, MD = −0.59, 95% CI (−0.86, −0.32), *P* < .0001) for significant heterogeneous distribution (*P* < .00001, I^2^ = 99%). The results of subgroup analysis showed that the combination of different courses of treatment was better than the control group in reducing 24h-UTP (2W: MD = −0.78, 95% CI (−1.02, −0.53), *P* < .00001; 4W: MD = −0.46, 95% CI (−0.74, −0.19), *P* = .001) (Fig. 8).

##### 3.4.2.4. mALB.

**Figure 9.**
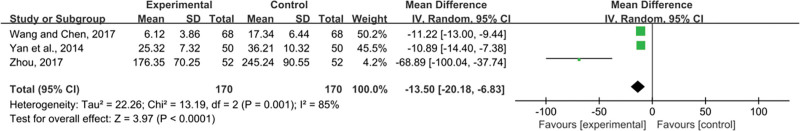
Effects of SML combined with ACEI/ARB vs ACEI/ARB on mALB. SML = salvia miltiorrhiza and ligustrazine.

In 3 investigations, mALB was mentioned. Pooled data were analyzed for substantial heterogeneous distribution (*P* = .001, I^2^ = 85%) using random-effects models (n = 340, MD = −13.50, 95% CI (−20.18, −6.83), *P* < .0001). According to the findings, combining SML with ACEI/ARB might considerably lower mALB when compared to the control group receiving ACEI/ARB alone (Fig. 9).

#### 3.4.3. Effects on blood glucose.

##### 3.4.3.1. 2hPBG.

**Figure 10.**
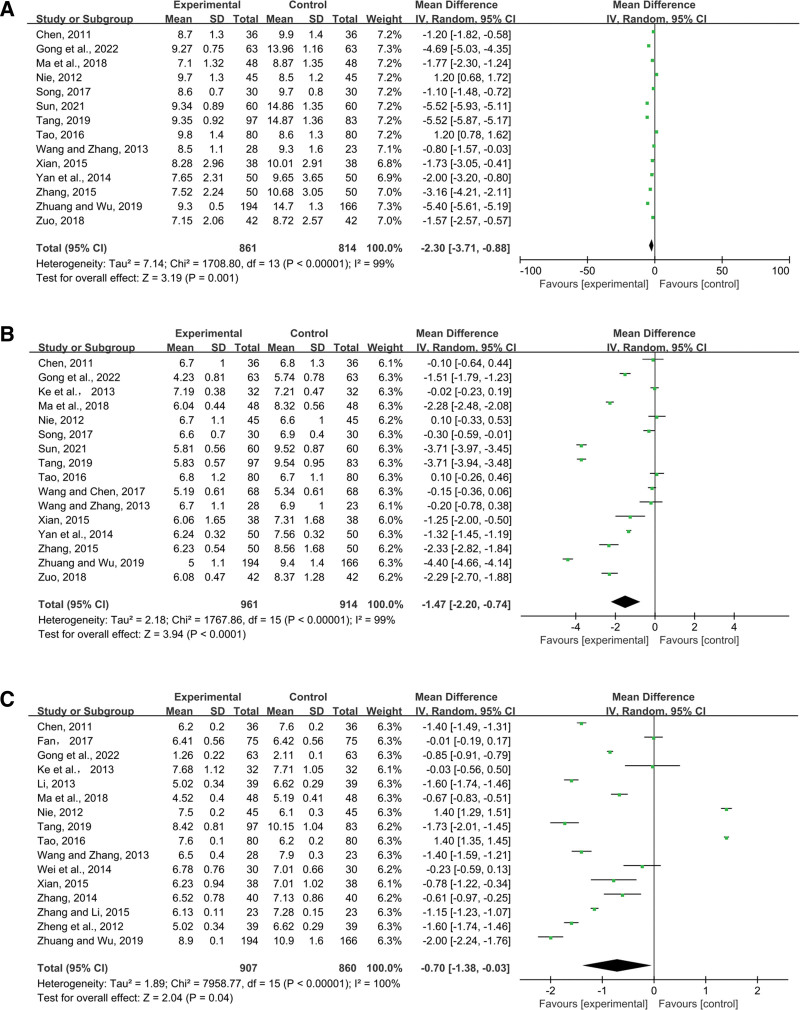
Effects of SML combined with ACEI/ARB vs ACEI/ARB on (A) 2hPBG, (B) FPG, (C) HbA1c. SML = salvia miltiorrhiza and ligustrazine.

Fourteen studies documented the use of 2hPBG. Pooled data (n = 1675, MD = 2.30, 95% CI (3.71, 0.88), *P* = .001) were analyzed for substantial heterogeneous distribution (*P* < .00001, I^2^ = 99%) using random-effects models. The findings demonstrated that 2hPBG was lowered more by the intervention group than by the control group (Fig. 10A).

##### 3.4.3.2. FPG.

The result FPG was present in 16 records. Pooled data were analyzed for substantial heterogeneous distribution (*P* < .00001, I^2^ = 99%) using random-effects models (n = 1875, MD = −1.47, 95% CI (−2.20, −0.74), *P* < .0001). According to a meta-analysis, SML in combination with ACEI/ARB reduced FPG better than ACEI/ARB alone (Fig. 10B).

##### 3.4.3.3. HbA1c.

In 16 investigations, HbA1c was mentioned. Pooled data were analyzed for significant heterogeneous distribution (*P* < .00001, I^2^ = 100%) using random-effects models (n = 1767 MD = −0.70, 95% CI (−1.38, −0.03), *P* = .04). As a consequence, the combination medicine group performed better than the control group in terms of HbA1c, according to the data (Fig. 10C).

#### 3.4.4. Effects on blood pressure.

##### 3.4.4.1. SBP.

**Figure 11.**
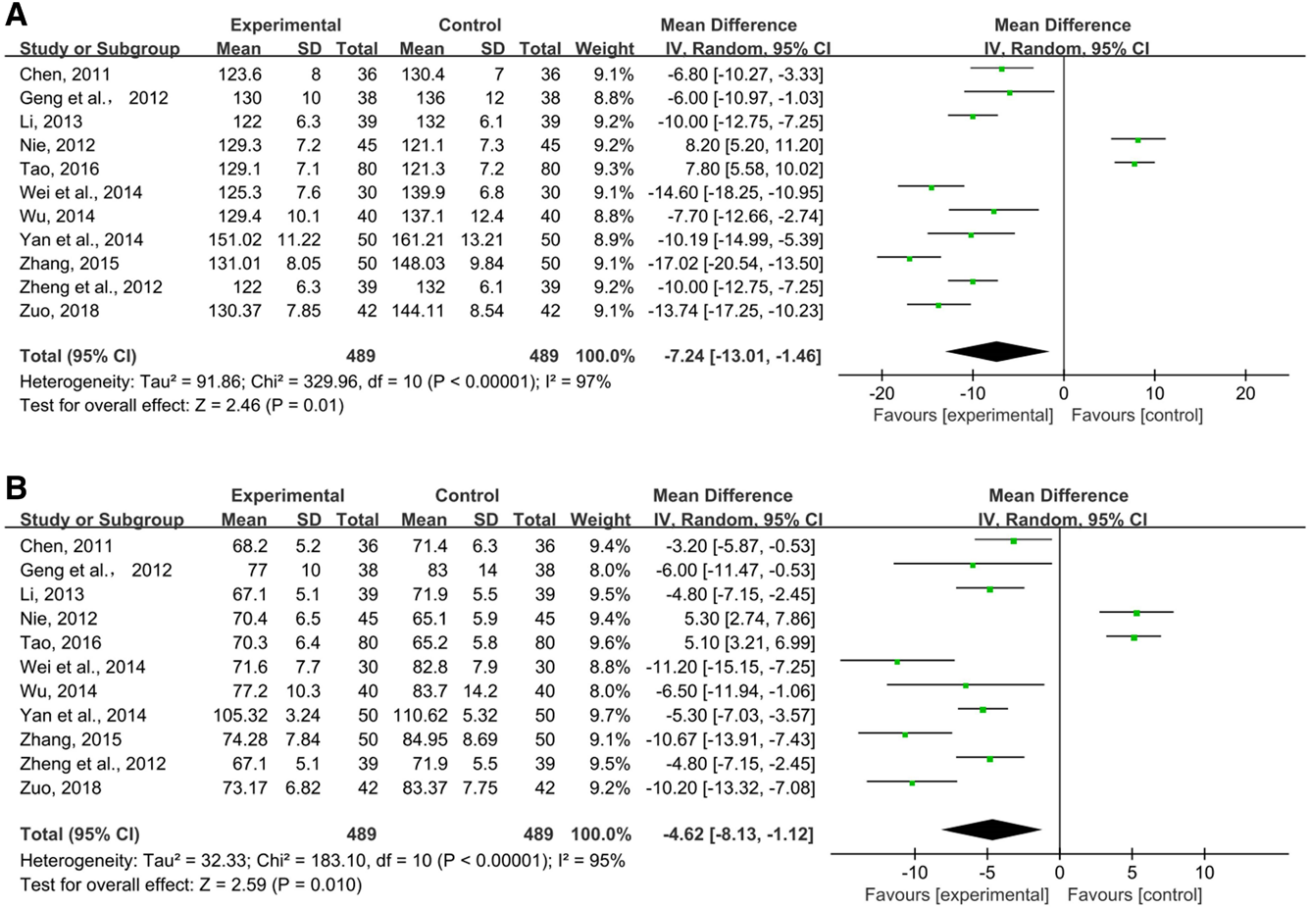
Effects of SML combined with ACEI/ARB vs ACEI/ARB on (A) SBP, (B) DBP. SML = salvia miltiorrhiza and ligustrazine.

In 11 studies, SBP was recorded. In order to detect significant heterogeneous distribution (*P* < .00001, I^2^ = 97%), random-effects models were used to pooled data (n = 978 MD = −7.24, 95% CI (−13.01, −1.46), *P* = .01). Combining treatments significantly reduced SBP, according to meta-analysis (Fig. 11A).

##### 3.4.4.2. DBP.

Eleven trials reported DBP. Random-effects models were used to analyze pooled data (n = 978 MD = −4.62, 95% CI (−8.13, −1.12), *P* = .01) for significant heterogeneous distribution (*P* < .00001, I^2^ = 95%). The results of Meta-analysis demonstrated that combined treatment was more significant in reducing DBP (Fig. 11B).

#### 3.4.5. Effects on blood lipids.

##### 3.4.5.1. TG.

**Figure 12.**
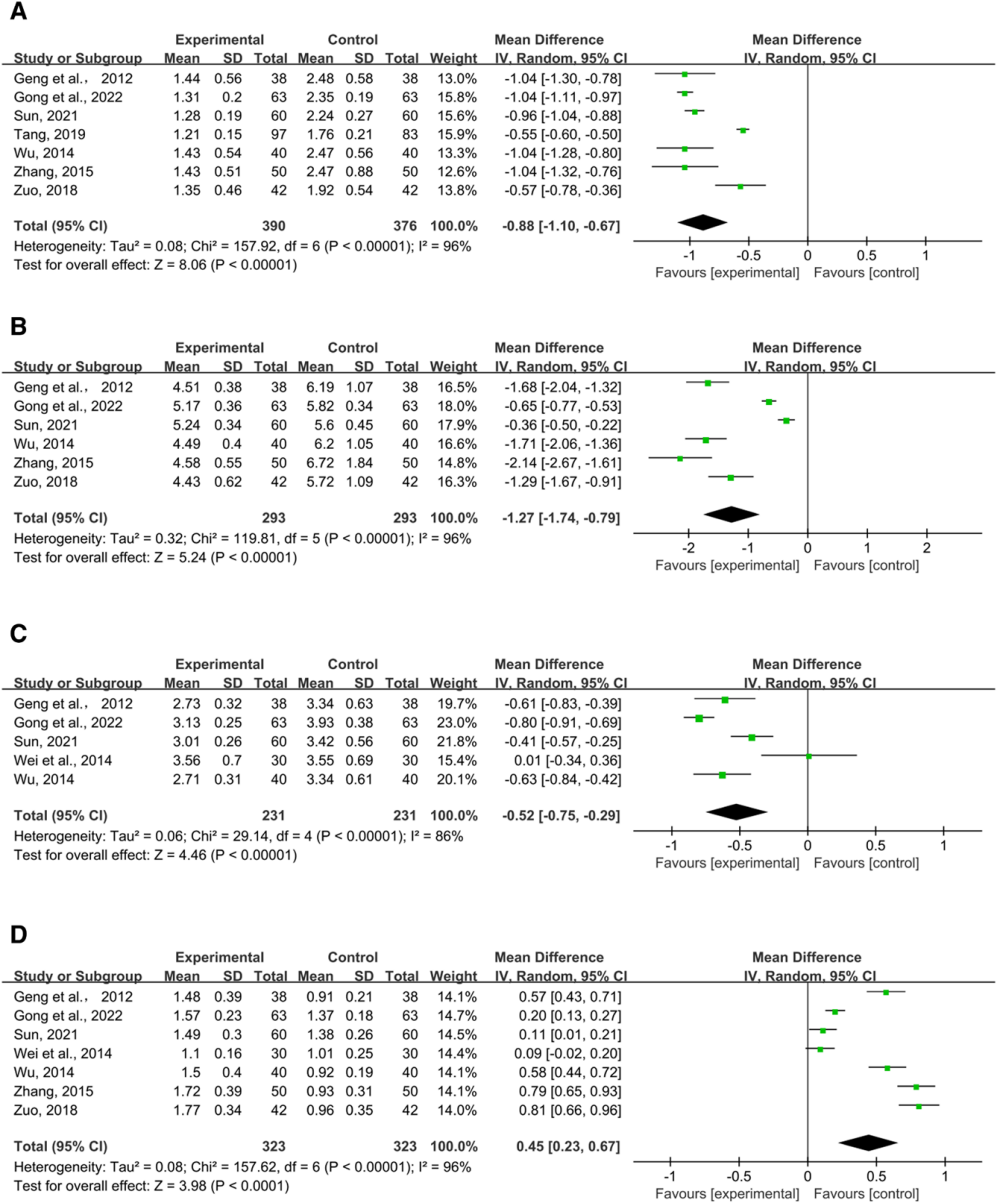
Effects of SML combined with ACEI/ARB vs ACEI/ARB on (A) TG, (B) TC, (C) LDL-C, (D) HDL-C. SML = salvia miltiorrhiza and ligustrazine.

Seven studies mentioned TG. Pooled data (n = 766 MD = −0.88, 95% CI (−1.10, −0.67), *P* < .00001) were analyzed for substantial heterogeneous distribution (*P* < .00001, I^2^ = 96%) using random-effects models. The findings showed that combination therapy was more effective in lowering TG (Fig. 12A).

##### 3.4.5.2. TC.

Six studies included TC reports. In order to detect significant heterogeneous distribution (*P *< .00001, I^2^ = 96%), pooled data (n = 586 MD = −1.27, 95% CI (−1.74, −0.79), *P* < .00001) were analyzed using random-effects models. The findings demonstrated that the combined approach was more effective in lowering TC (Fig. 12B).

##### 3.4.5.3. LDL-C.

Five studies included the outcome LDL-C. Random-effects models were used to analyze pooled data (n = 462, MD = −0.52, 95% CI (−0.75, −0.29), *P* < .00001) for significant heterogeneous distribution (*P* < .00001, I^2^ = 86%). Meta-analysis showed that combined intervention was more advantageous than ACEI/ARB in reducing LDL-C (Fig. 12C).

##### 3.4.5.4. HDL-C.

The result of HDL-C was reported by 7 research. Pooled data were analyzed for substantial heterogeneous distribution (*P* < .00001, I^2^ = 96%) using random-effects models (n = 646, MD = 0.45, 95% CI (0.23, 0.67), *P* < .0001). In terms of controlling HDL-C, meta-analysis revealed that combined intervention outperformed the control group (Fig. 12D).

#### 3.4.6. Effects on inflammatory factors.

##### 3.4.6.1. IL-6.

**Figure 13.**
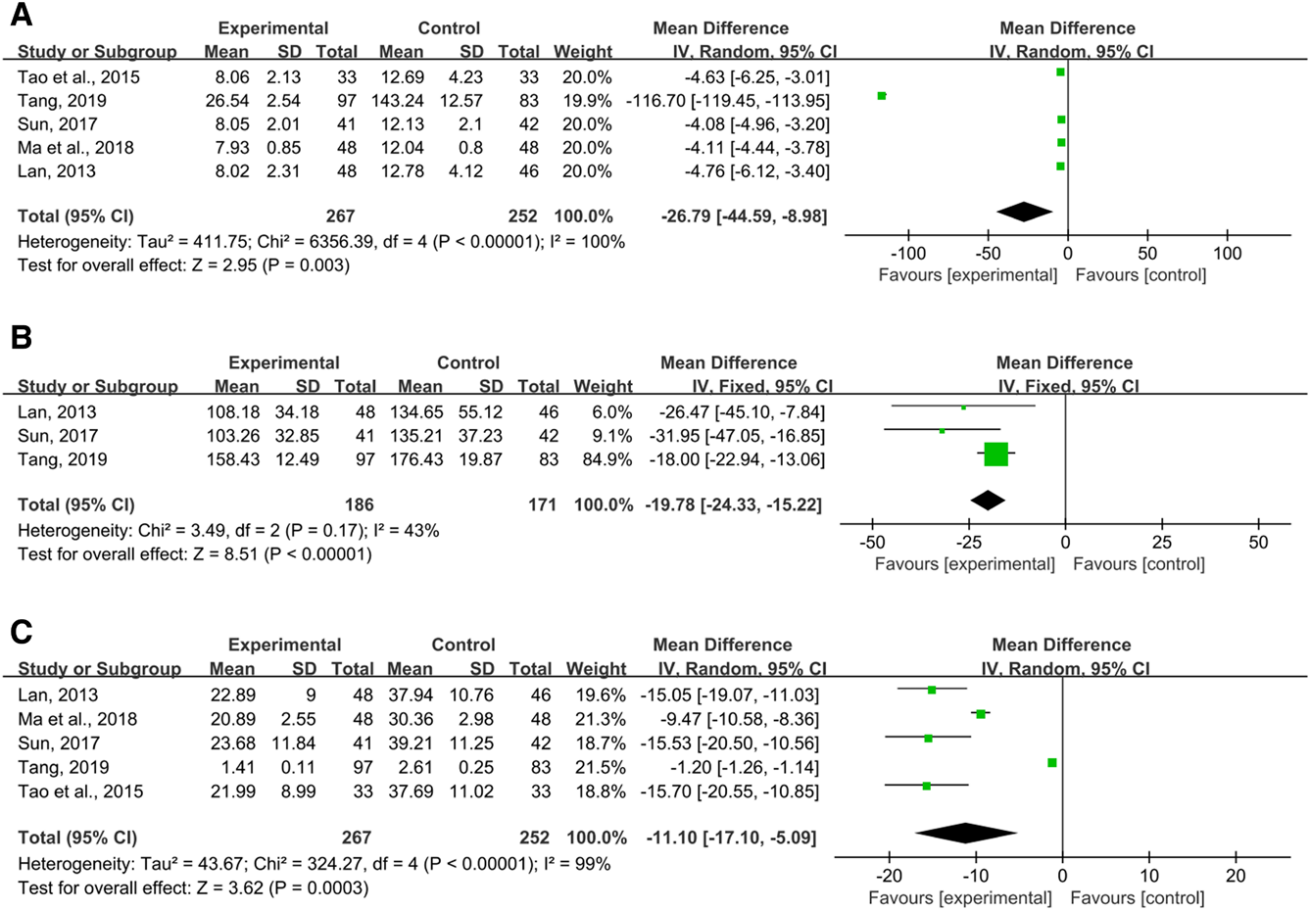
Effects of SML combined with ACEI/ARB vs ACEI/ARB on (A) IL-6, (B) IL-18, (C) TNF-α. SML = salvia miltiorrhiza and ligustrazine.

Five records included the outcome IL-6. Random-effects models were used to analyze pooled data (n = 519 MD = −26.79, 95% CI (−44.59, −8.98), *P* = .003) for significant heterogeneous distribution (*P* < .00001, I^2^ = 100%), and the results showed that the IL-6 of the 2 groups had a statistical difference, and the combined medication group was better than the control group (Fig. 13A).

##### 3.4.6.2. IL-18.

A total of 3 papers mentioned IL-18. Pooled data were analyzed using fixed-effects models (n = 357 MD = −19.78, 95% CI (−24.33, −15.22), *P* < .00001). No discernible heterogeneous distribution was found (*P* = .17, I^2^ = 43%) when the homogeneity statistic was examined. The outcomes showed that SML in combination with ACEI/ARB could considerably lower IL-18 in comparison to the control group with traditional ACEI/ARB alone (Fig. 13B).

##### 3.4.6.3. TNF-α.

Five studies reported the outcome TNF-α. Random-effects models were used to analyze pooled data (n = 519 MD = −11.10, 95% CI (−17.10, −5.09), *P* = .0003) for significant heterogeneous distribution (*P* < .00001, I^2^ = 99%). The results showed that the TNF-α of the 2 groups had a statistical difference, and the combined ACEI/ARB was better than the control group (Fig. 13C).

### 3.5. Adverse events

Eleven studies reported detailed adverse events. The clinical manifestations of adverse events were dry cough, pruritus, skin rash, constipation, insomnia, edema, headaches, dizziness, painful blood vessels at the injection site, fatigue, diarrhea, postural hypotension and others (not specified in text). No subjects discontinued the drug. A summary of adverse events is presented in Table 2.

**Table 2:** Percentage of different adverse events.

Adverse events	SML with ACEI/ARB (659 patients)	ACEI/ARB (651 patients)
Dry cough	15 (2.28%)	23 (3.53%)
Pruritus	7 (1.06%)	8 (1.23%)
Skin rash	4 (0.61%)	0 (0.00%)
Constipation	1 (0.15%)	1 (0.15%)
Insomnia	0 (0.00%)	2 (0.31%)
Edema	0 (0.00%)	3 (0.46%)
Headaches	7 (1.06%)	7 (1.08%)
Dizziness	4 (0.61%)	5 (0.77%)
Painful blood vessels at the injection site	3 (0.46%)	0 (0.00%)
Fatigue	0 (0.00%)	5 (0.77%)
Diarrhea	0 (0.00%)	5 (0.77%)
Postural hypotension	4 (0.61%)	5 (0.77%)
Others (not specified in text)	1 (0.15%)	0 (0.00%)

### 3.6. Subgroup analysis

Subgroup analyses were performed according to different strata of control (ARB or ACEI) and intervention time (2W, 3W, 4W, 12W, 16W) to investigate the effect of SML on renal function and proteinuria indexes. Supplementary material-1, http://links.lww.com/MD/L668 show in detail the subgroup analyses for the different control groups and the subgroup analyses for the different intervention times are shown in the results above.

The subgroup analysis showed that SML either combined with ARB or ACEI treatment significantly improved renal function and proteinuria indexes compared to the control group, and the improvement of Scr, BUN and 24h-UTP indexes was more significant with combined ACEI treatment compared to ARB, while for UAER improvement, the results were more significant with combined ARB treatment than combined ACEI treatment. In terms of intervention duration, the combination of SML with 2 weeks of intervention significantly improved SCr, BUN, UAER, and 24-UTP, while no significant improvement in SCr and BUN was observed at 3 or 16 weeks of intervention.

### 3.7. Publication bias and sensitivity

**Figure 14.**
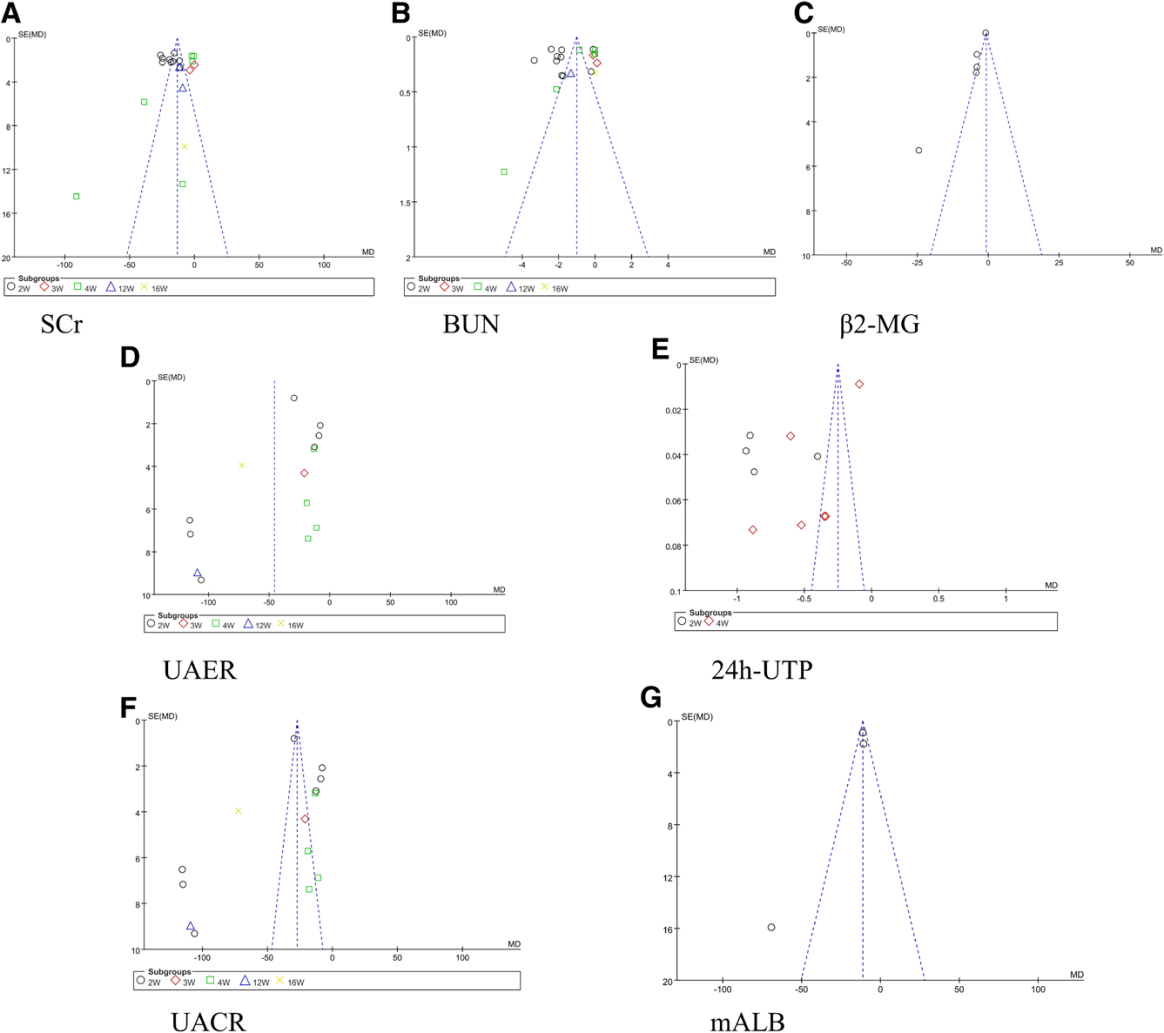
Funnel plot (A) Scr, (B) BUN, (C) β2-MG, (D) UAER, (E) 24h-UTP, (F) UACR, (G) mALB.

Funnel plots were used to assess publication bias in renal function and urinary protein indicators (Fig. 14). Egger ‘s test *P* values for SCr, BUN, UAER, UACR, mALB, 2hPBG, FPG, HbA1c, SBP, DBP, TG, LDL-C, IL-6 and IL-18 were all >0.05, and no publication bias was observed. *P* values for β2-MG, 24h-UTP, TC, HDL-C and TNF-α were all <0.05, suggesting a possible publication bias for these results. Details of the Egger test results are presented in Table 3.

**Table 3:** Publication bias based on Egger tests.

Primary outcome	*P* value
SCr	.673
BUN	.469
β2-MG	.017
UAER	.232
UACR	.206
24h-UTP	.009
mALB	.309
2hPBG	.060
FPG	.928
HbA1c	.157
SBP	.112
DBP	.209
TG	.491
TC	.012
LDL-C	.145
HDL-C	.033
IL-6	.297
IL-18	.257
TNF-α	.035

Sensitivity analysis showed that the results were relatively stable except for IL-6, IL-18 and mALB. The results of sensitivity analysis are shown in the supplementary material-2, http://links.lww.com/MD/L669. We found that heterogeneity decreased from 100%, 43% to 0% in both IL-6 and IL-18 evidence bodies after removal of the test (Tang, 2019), respectively. This may be due to the fact that this test had the largest effect size and was the only test with statistically significant differences between the 2 groups. In the meta-analysis of mALB, heterogeneity then decreased from 99% to 0% after the exclusion trial (Zhou, 2017). Heterogeneity may arise from the different disease stages in the study. See supplementary material-3, http://links.lww.com/MD/L670 for details.

## 4. Discussion

In recent years, salvia miltiorrhiza, its main active ingredients and its compound preparations also play a positive role in the treatment of DKD, which can regulate abnormal glucose and lipid metabolism, anti-oxidative stress response, reduce inflammatory reactions such as cytokines and signaling pathways, improve renal blood flow, and even improve gene metabolism, so as to protect renal function and improve glomerular filtration ability. Thus, the stability of blood sugar is maintained again, forming a virtuous cycle and playing a role in alleviating or even reversing the process of DKD.^[52]^ Based on the meta-analysis of this review, we provided an updated investigation of the efficacy and safety of SML injection as the treatment for DKD.

### 4.1. Summary of evidence

Based on the results of the current systematic review, efficacy of SML injection combined with or without ACEI/ARB in the treatment of DKD were evaluated. This systematic review identified 30 RCTs containing 3214 patients for analysis.

The results of meta-analysis showed that the combined application of SML injection on the basis of ACEI/ARB had better clinical efficacy in the treatment of DKD, which could improve renal function more effectively and reduce the levels of renal albuminuria. The combination of the 2 also showed some efficacy in lowering the levels of blood glucose (2hPBG, FPG, HbA1c), blood lipids (TG, TC, LDL-C, HDL-C), blood pressure (SBP, DBP) and inflammatory factors (IL-6, IL-18, TNF-α).

For safety evaluation, there are no post-marketing surveillance studies of SML injection. It is preliminarily believed that SML injection is safe for adjuvant treatment of DKD. However, some studies have not reported the safety indexes of SML injection. Although adverse reactions have been reported in the experimental group, the results obtained are not completely accurate considering the combination of ACEI/ARB and the lack of evidence on whether SML injection is directly related to symptoms. And it should be noted that this dose range is not supported by long-term data. Therefore, more specific security assessments are needed as evidence for further discussion.

The results of pharmacological experiments show that the active ingredients of Salvia miltiorrhiza can improve microcirculation, dilate blood vessels, protect kidney function, and have a certain effect on the prevention and treatment of DKD; ligustrazine is a calcium antagonist, which can improve microcirculation, antiplatelet aggregation, reduce red blood cell viscosity, inhibit microinflammatory reaction in diabetic patients, and has a protective effect on kidney function, so its application in the treatment of DKD has a sufficient theoretical basis and practical foundation.^[56]^ Here, our study is consistent with published findings and further confirms the effectiveness of SML injection combined with ACEI/ARB therapy. Compared with ACEI/ARB, Danshen and chasteberry injection combined with ACEI/ARB in treating DKD patients can significantly improve the main indexes related to renal function, such as UAER, UACR, 24h-UTP, mALB, Scr, BUN, and β2-MG. However, given the uncertain risks of the included studies, the evidence needs to be further validated. Furthermore, the small sample sizes of UACR (3 studies, 243 individuals), mALB (3 studies, 240 individuals), and IL-18 (3 studies, 357 individuals) may lead to higher variability, which in turn may lead to bias and affect the reliability of the results. In addition to the significant improvement in efficacy, the results of adverse reactions demonstrated a better safety profile.

After a literature review, it was found that SML injection has been widely used in the treatment of cardiovascular diseases, including coronary artery disease, angina pectoris and atherosclerosis, by down-regulating pro-apoptotic factors, ameliorating inflammation and correcting dyslipidemia.^[53,54]^ However, the evidence for clinical application in DKD patients remains unclear. After a systematic review and meta-analysis, we further concluded that the combination of SML injection with ACEI/ARB in patients with DKD improves renal function, nephrogenic proteinuria, hyperglycemia, blood pressure, dyslipidemia, and inflammatory response with a high degree of safety.

### 4.2. Research limitations

The review has some inherent and methodological limitations. First, we only searched databases in Chinese and English, with no other languages available, which may have resulted in language bias. In addition, the 30 included clinical trials were published in Chinese journals, which limits the wide application of the data. Second, although the included trials were randomized controlled trials, the random sequence generation and blinding of the included trials were generally standardized and rigorously implemented, which also reduces the certainty of the evidence. Third, almost all of the included trials lacked blinding, and different combined interventions between the 2 groups could lead to bias. There are some concerns about the risk of bias, which are reflected in the Cochrane risk of bias tool ratings of the quality of evidence. Fourth, all included trials were conducted in China, which may limit the generalization of results. Fifth, there are several double-blind randomized clinical trials currently underway, so this systematic review is incomplete and will be updated. Finally, some may argue that the methodological quality of published trials in TCM is lower than in other fields, which greatly hinders the systematic evaluation process. However, we prefer to take a positive and optimistic approach and identify problems and shortcomings through the current review to provide a basis for further research.

We found that the included studies reported inconsistent results, which prevented researchers from conducting meta-analyses. This will significantly reduce the value of research and cause a waste of resources. Outcomes of future studies in this area should be selected according to the DKD core outcome set, including decreased renal function, increased risk of cardiovascular disease, and progression to renal failure requiring dialysis or transplantation. Some studies have yet to report significant or long-term results, which is not conducive to evaluating the primary efficacy and long-term benefits of SML injection. Therefore, future DKD studies should evaluate the long-term effects of SML injection, such as cardiovascular events (heart failure, myocardial infarction, cardiovascular death) and kidney disease progression. In addition, researchers should rigorously design trials concerning criteria such as the Standard Protocol Project: Interventional trial recommendations, including appropriate allocation concealment and blind methods, and more attention should be paid to the choice of outcome measurement tools and statistical methods.

Finally, researchers cannot neglect to evaluate the interaction effects of SML injection in combination with conventional drugs.

## 5. Conclusions

In individuals with DKD, we discovered that coupled ACEI/ARB may improve renal function, proteinuria, glucose dyslipidemia, and even blood pressure and inflammation. However, the development of renal disease and cardiovascular events are not presently being evaluated in any research. In order to investigate the long-term effects of SML injection, future research should go beyond surrogate endpoints to actual cardiovascular or renal prognostic advantages.

## Acknowledgments

Thanks to Prof Zhong and all the authors for their assistance in writing this manuscript.

## Author contributions

**Conceptualization:** Zixuan Zhang.

**Data curation:** Zixuan Zhang, Lei Luo, Xueling Li.

**Investigation:** Lei Luo, Xueling Li.

**Methodology:** Zixuan Zhang.

**Software:** Zixuan Zhang.

**Writing – original draft:** Zixuan Zhang, Lei Luo.

**Writing – review & editing:** Yifei Zhong.

## Supplementary Material









## References

[1] Zhang T Zhang Q Zheng W. Fructus Zanthoxyli extract improves glycolipid metabolism disorder of type 2 diabetes mellitus via activation of AMPK/PI3K/Akt pathway: network pharmacology and experimental validation. J Integr Med. 2022;20:543–60. 35965234 10.1016/j.joim.2022.07.004

[2] Tan SY Mei Wong JL Sim YJ. Type 1 and 2 diabetes mellitus: a review on current treatment approach and gene therapy as potential intervention. Diabetes Metab Syndr. 2019;13:364–72. 30641727 10.1016/j.dsx.2018.10.008

[3] Sun H Saeedi P Karuranga S. IDF diabetes atlas: global, regional and country-level diabetes prevalence estimates for 2021 and projections for 2045. Diabetes Res Clin Pract. 2022;183:109119. 34879977 10.1016/j.diabres.2021.109119 PMC11057359

[4] Johansen KL Chertow GM Gilbertson DT. US renal data system 2021 annual data report: epidemiology of kidney disease in the United States. Am J Kidney Dis. 2022;79:A8–A12. 35331382 10.1053/j.ajkd.2022.02.001 PMC8935019

[5] Cole Joanne B Florez Jose C. Genetics of diabetes mellitus and diabetes complications. Nat Rev Nephrol. 2020;16:377–90. 32398868 10.1038/s41581-020-0278-5 PMC9639302

[6] Cheng HT Xu X Lim PS. Worldwide epidemiology of diabetes-related end-stage renal disease, 2000-2015. Diabetes Care. 2021;44:89–97. 33203706 10.2337/dc20-1913

[7] Jiang W Wang J Shen X. Establishment and validation of a risk prediction model for early diabetic kidney disease based on a systematic review and meta-analysis of 20 cohorts. Diabetes Care. 2020;43:925–33. 32198286 10.2337/dc19-1897

[8] Alicic RZ Rooney MT Tuttle KR. Diabetic kidney disease: challenges, progress, and possibilities. Clin J Am Soc Nephrol. 2017;12:2032–45. 28522654 10.2215/CJN.11491116 PMC5718284

[9] Tonneijck L Muskiet MH Smits MM. Glomerular hyperfiltration in diabetes: mechanisms, clinical significance, and treatment. J Am Soc Nephrol. 2017;28:1023–39. 28143897 10.1681/ASN.2016060666 PMC5373460

[10] de Boer IH Caramori ML Chan JCN. Executive summary of the 2020 KDIGO diabetes management in CKD guideline: evidence-based advances in monitoring and treatment. Kidney Int. 2020;98:839–48. 32653403 10.1016/j.kint.2020.06.024

[11] McGrath K Edi R. Diabetic kidney disease: diagnosis, treatment, and prevention. Am Fam Physician. 2019;99:751–9. 31194487

[12] He D Zhang Y Zhang W. Effects of ACE inhibitors and angiotensin receptor blockers in normotensive patients with diabetic kidney disease. Horm Metab Res. 2020;52:289–97. 32219798 10.1055/a-1138-0959

[13] Sugahara M Pak WLW Tanaka T. Update on diagnosis, pathophysiology, and management of diabetic kidney disease. Nephrology (Carlton). 2021;26:491–500. 33550672 10.1111/nep.13860

[14] Whitlock R Leon SJ Manacsa H. The association between dual RAAS inhibition and risk of acute kidney injury and hyperkalemia in patients with diabetic kidney disease: a systematic review and meta-analysis. Nephrol Dial Transplant. 2023;38. 10.1093/ndt/gfad101 PMC10615629 37309038

[15] Gosmanova EO Molnar MZ Naseer A. Longer predialysis ACEi/ARB utilization is associated with reduced postdialysis mortality. Am J Med. 2020;133:1065–1073.e3. 32330490 10.1016/j.amjmed.2020.03.037 PMC7483641

[16] Picard M. Why do we care more about disease than health? Phenomics. 2022;2:145–55. 36939781 10.1007/s43657-021-00037-8 PMC9590501

[17] Sun H Guo YS Zhang M. Clinical effect of Salvia miltiorrhiza and ligustrazine on early diabetic nephropathy and its effect on inflammatory factors. J Beihua Univ Nat Sci. 2017;8:478–82.

[18] Pei JM Li RF Cheng G. Clinical evaluation of Salvia miltiorrhiza and ligustrazine injection in the treatment of diabetic nephropathy. Chin J Clin Pharmacol. 2016;32:1507–11.

[19] Wang H Sun Y Ma Q. Exploring the targets and mechanisms of Salvia miltiorrhiza in the treatment of diabetic nephropathy based on network pharmacology and diabetic nephropathy rat model. J Xuzhou Med Univ. 2022;42:859–65.

[20] Pei JM Li RF Cheng G. Clinical evaluation of Salvia miltiorrhiza and ligustrazine injection in the treatment of diabetic nephropathy. Chin J Clin Pharmacol. 2016;32:1507–11.

[21] Xie F Zhang B Dai S. Efficacy and safety of Salvia miltiorrhiza (Salvia miltiorrhiza Bunge) and ligustrazine injection in the adjuvant treatment of early-stage diabetic kidney disease: a systematic review and meta-analysis. J Ethnopharmacol. 2021;281:114346. 34153447 10.1016/j.jep.2021.114346

[22] Gong J Li X Zhang F. Clinical efficacy of Danshen chuanxiongzin injection combined with western medicine in the treatment of diabetic nephropathy and its effects on renal function and blood glucose and lipids. J Pract Chin Med Intern Med. 2022;36:64–6.

[23] Sun D. Effect of Danshen chuanxiongzin combined with Fosinopril in the treatment of patients with diabetic nephropathy. Chin Min Kang Med. 2021;33:96–8.

[24] Tang N. Clinical efficacy of Danshen chuanxiongzin injection combined with Fosinopril in patients with diabetic nephropathy with proteinuria. Chin Patent Med. 2019;41:559–62.

[25] Zhuang G Wu X. The value of Danshen chuanxiongzin injection combined with Fosinopril in diabetic nephropathy. Henan Med Res. 2019;28:4137–8.

[26] Zuo AF Li M. Clinical effects of the combination of Irbesartan and Danshen chuanxiongzin in the treatment of diabetic nephropathy. J Clin Med Res Pract. 2018;3:41–2.

[27] Ma X Zhu GZ Zhu Q. Clinical observation of Irbesartan combined with Danshen chuanxiongzin in the treatment of diabetic nephropathy. Gansu Med. 2018;37:605–6.

[28] Feng H. Analysis of the effect of Danshen chuanxiongzin combined with Benadryl in the treatment of early type 2 diabetic nephropathy. Henan Med Res. 2018;27:2970–2.

[29] Sun H Guo YS Zhang M. Clinical effects of Danshen chuanxiongzin in the treatment of early diabetic nephropathy and its effect on inflammatory factors. J Beihua Univ (Nat Sci Ed). 2017;18:478–82.

[30] Song X. Clinical effect study of Danshen chuanxiongzin in the treatment of early type 2 diabetic nephropathy. Diabetes New World. 2017;20:168–9.

[31] Fan W. Observation on the effect of valsartan combined with danshen chuanxiongzin injection in the treatment of microproteinuria in type 2 diabetes mellitus. China Mod Drug Appl. 2017;11:107–8.

[32] Wang Y Chen LY. Clinical observation on the treatment of early diabetic nephropathy with Danshen chuanxiongzin injection supplemented with Benadryl. Zhejiang Pract Med. 2017;22:337–9.

[33] Zhou CM. Observation and evaluation of the effect of applying Danshen chuanxiongzin in the treatment of diabetic nephropathy. Psychologist. 2017;23:156.

[34] Tao Y. Clinical efficacy of Danshen chuanxiongzin injection in the treatment of early diabetic nephropathy. Diabetes New World. 2016;19:15–6.

[35] Zhang L Li Y. Observation on the efficacy of Danshen chuanxiongzin injection combined with western medicine in the treatment of early diabetic nephropathy. China Mod Drug Appl. 2015;9:142–3.

[36] Line LH. 38 cases of early diabetic nephropathy treated with Danshen chuanxiongzin injection combined with valsartan. China Pharm. 2015;24:95–6.

[37] Tao L Tong J Cao L. Clinical observation of 33 cases of early diabetic nephropathy treated with Danshen chuanxiongzin injection. New Chin Med. 2015;47:90–1.

[38] Zhang HT. Clinical analysis of Irbesartan combined with Danshen chuanxiongzin in the treatment of diabetic nephropathy. Chin Foreign Med Res. 2015;13:114–5.

[39] Pan R. Clinical observation of 72 cases of diabetic nephropathy stage IV treated with Danshen chuanxiongzin injection combined with conventional therapy. J Henan Univ Sci Technol (Med Ed). 2015;33:200–2.

[40] Zhang Y. Clinical observation on the combination of Danshen chuanxiongzin injection and Cloxacin in the treatment of early diabetic nephropathy. Diabetes New World. 2014;34:23.

[41] Yan CJ Zhang DB Ye SR. Observation on the effect of treatment of diabetic nephropathy with Irbesartan combined with Danshen Chuanxiongzin. New World Diabetes. 2014;34:25–6.

[42] Wei XP Wang XZ Wu Z. Clinical observation on the treatment of diabetic nephropathy with temisartan combined with danshen chuanxiongzin. China Pract Med. 2014;9:1–2.

[43] Wu LH. Discussion on the effect of blood pressure and lipids after treatment of diabetic nephropathy with Irbesartan combined with Danshen Chuanxiongzin. Knowl Cardiovasc Dis Control (Acad Ed). 2014;22:28–30.

[44] Wang SL Zhang Y. Analysis of the efficacy of valsartan combined with danshen chuanxiongzin in the treatment of early diabetic nephropathy. China Prim Med. 2013;20:1485–6.

[45] Ke XY Shi YQ Li MX. Effect of danshen chuanxiongzin injection combined with valsartan on microproteinuria in early diabetic nephropathy. Shi Zhen Guo Yi Guo Yao. 2013;24:1235–6.

[46] Lan Z. Efficacy of Danshen Chuanxiongzin in the treatment of early diabetic nephropathy and the effect on inflammatory factors. Shi Zhen Guo Yi Guo Yao. 2013;24:1693–4.

[47] Li Y. Analysis of the efficacy of valsartan combined with danshen chuanxiongzin injection in the treatment of early diabetic nephropathy. Med Inf. 2013;9:473.

[48] Nie M. Clinical observation on the treatment of early diabetic nephropathy with Danshen and Chuanxiongzin injection combined with Cloxacin. Chin Min Kang Med. 2012;24:28–9 + 31.

[49] Zheng DJ Hu Q Huang J. Clinical observation of Danshen Chuanxiongzin injection combined with valsartan in the treatment of early diabetic nephropathy. Chinese J Mod Med. 2012;22:83–4.

[50] Geng M Ren GY Wang SY. Observation of Irbesartan combined with Danshen Chuanxiongzin in the treatment of diabetic nephropathy. Chin Pharm. 2012;15:1761–3.

[51] Chen G. Observation of 36 cases of early diabetic nephropathy treated with Danshen Chuanxiongzin injection combined with valsartan. Chin Community Physicians (Med Specialties). 2011;13:171–2.

[52] Yu H Yu HY Mou AM. Research progress on the protective mechanism of Salvia miltiorrhiza against diabetic nephropathy. World Tradit Chin Med. 2021;16:1161–5.

[53] Huang W Yang Y Zeng Z. Effect of Salvia miltiorrhiza and ligustrazine injection on myocardial ischemia/reperfusion and hypoxia/reoxygenation injury. Mol Med Rep. 2016;14:4537–44. 27748867 10.3892/mmr.2016.5822 PMC5101990

[54] Zhao X Chen Y Li L. Effect of DLT-SML on chronic stable angina through ameliorating inflammation, correcting dyslipidemia, and regulating gut microbiota. J Cardiovasc Pharmacol. 2021;77:458–69. 33657052 10.1097/FJC.0000000000000970

[55] Li X. Clinical applications of chuanxiong and its extracts. Gansu Med. 2017;36:344–6.

