# Supplementary material for: The effects of Salvia miltiorrhiza and ligustrazine injection combined with ACEI/ARB on diabetic kidney disease: A systematic review and meta-analysis
Source: Medicine (Baltimore). 2024 Feb 23;103(8):e35853. doi: 10.1097/MD.0000000000035853 (PMC11309681; doi:10.1097/MD.0000000000035853)
Supplement: Supplementary file 1 [file medi-103-e35853-s001.docx]

**Table S1：Search strategy for databases.**

| Databases | Elements | Search detail |
| --- | --- | --- |
| PubMed | Population | "Diabetic Nephropathies"[MeSH Terms] OR "Nephropathies, Diabetic"[Title/Abstract] OR "Nephropathy, Diabetic"[Title/Abstract] OR "Diabetic Nephropathy"[Title/Abstract] OR "Diabetic Kidney Disease"[Title/Abstract] OR "Diabetic Kidney Diseases"[Title/Abstract] OR "Kidney Disease, Diabetic"[Title/Abstract] OR "Kidney Diseases, Diabetic"[Title/Abstract] OR "Diabetic Glomerulosclerosis"[Title/Abstract] OR "Glomerulosclerosis, Diabetic"[Title/Abstract] OR "Intracapillary Glomerulosclerosis"[Title/Abstract] OR "Nodular Glomerulosclerosis"[Title/Abstract] OR "Glomerulosclerosis, Nodular"[Title/Abstract] OR "Kimmelstiel-Wilson Syndrome"[Title/Abstract] OR "Kimmelstiel Wilson Syndrome"[Title/Abstract] OR "Syndrome, Kimmelstiel-Wilson"[Title/Abstract] OR "Kimmelstiel-Wilson Disease"[Title/Abstract] OR "Kimmelstiel Wilson Disease"[Title/Abstract] OR "DKD"[Title/Abstract] OR "DN"[Title/Abstract] |
|  | Intervention | "Salvia miltiorrhiza"[MeSH Terms] OR "Tan Seng"[Title/Abstract] OR "Dan-Shen"[Title/Abstract] OR "Dan Shen"[Title/Abstract] OR "Chinese Salvia"[Title/Abstract] OR "Chinese Salvias"[Title/Abstract] OR "Salvia, Chinese"[Title/Abstract] OR "Salvias, Chinese"[Title/Abstract] OR "Salvia miltiorrhizae"[Title/Abstract] AND "tetramethylpyrazine"[MeSH Terms] OR "ligustrazine"[Title/Abstract] OR "TMPZ"[Title/Abstract] OR "chuanxiongzine"[Title/Abstract] OR "tetramethyl pyrazine"[Title/Abstract] OR "tetramethylpyrazine hydrochloride"[Title/Abstract] OR "Liqustrazine"[Title/Abstract] AND "Injections[MeSH Terms] OR "Injectables"[Title/Abstract] OR "Injection"[Title/Abstract] OR "Injectable"[Title/Abstract] OR "Salvia miltiorrhiza and ligustrazine"[Title/Abstract] OR "Danshenchuanxiongqin"[Title/Abstract] |
|  | Study Design | "Randomized Controlled Trial"[MeSH Terms] OR "RCT"[Title/Abstract] |
| Web of Science | Population | TS=(Diabetic Nephropathies) OR TS=(Diabetic Nephropathy) OR TS=(Diabetic Kidney Diseas) OR TS=(Diabetic Kidney Diseases) OR TS=(Diabetic Glomerulosclerosis) OR TS=(Intracapillary Glomerulosclerosis) OR TS=(Nodular Glomerulosclerosis) OR TS=(Kimmelstiel-Wilson Syndrome) OR TS=(Kimmelstiel Wilson Syndrome) OR TS=(Kimmelstiel-Wilson Disease) OR TS=(Kimmelstiel Wilson Disease) OR TS=(DKD) OR TS=(DN) |
|  | Intervention | (TS=(Salvia miltiorrhiza) OR TS=(Tan Seng) OR TS=(Dan Shen) OR TS=(Dan-Shen) OR TS=(Chinese Salvia) OR TS=(Chinese Salvias) OR TS=(Salvia miltiorrhizae)) AND (TS=(tetramethylpyrazine) OR TS=(ligustrazine) OR TS=(TMPZ) OR TS=(chuanxiongzine) OR TS=(chuanxiongqin) OR TS=(tetramethyl pyrazine) OR TS=(tetramethylpyrazine hydrochloride) OR TS=(Liqustrazine)) AND (TS=(Injections) OR TS=(Injectables) OR TS=(Injection) OR TS=(Injectable)) |
|  | Study Design | TS=(Randomized Controlled Trial) OR TS=(RCT) |
| CNKI | Population | (主题：糖尿病肾病) OR (主题：糖尿病性肾病) OR (主题：糖尿病肾小球硬化症) OR (主题：糖尿病微血管并发症) |
|  | Intervention | (((主题：丹参) AND (主题：盐酸川芎嗪)) OR (主题：丹参川芎嗪)) AND (主题：注射液) |
|  | Study Design | (主题：随机对照试验) OR (主题：RCT) |
| WanFang | Population | 主题:(糖尿病肾病) or 主题:(糖尿病性肾病) or 主题:(糖尿病肾小球硬化症) or 主题:(糖尿病微血管并发症) |
|  | Intervention | ((主题:(丹参) and 主题:(盐酸川芎嗪)) or 主题:(丹参川芎嗪)) and 主题:(注射液) |
|  | Study Design | 主题:(随机对照试验) or 主题:(RCT) |
| VIP | Population | 题名或关键词=糖尿病肾病 OR 题名或关键词=糖尿病性肾病 OR 题名或关键词=糖尿病肾小球硬化症 OR 题名或关键词=糖尿病微血管并发症 |
|  | Intervention | ((题名或关键词=丹参 AND 题名或关键词=盐酸川芎嗪) OR 题名或关键词=丹参川芎嗪) AND 题名或关键词=注射液 |
|  | Study Design | 题名或关键词=随机对照试验 OR 题名或关键词=RCT |
| SinoMed | Population | “糖尿病肾病”[常用字段：智能] OR “糖尿病性肾病”[常用字段：智能] OR “糖尿病肾小球硬化症”[常用字段：智能] OR “糖尿病微血管并发症”[常用字段：智能] |
|  | Intervention | ((“丹参”[常用字段：智能] AND “盐酸川芎嗪”[常用字段：智能]) OR “丹参川芎嗪”[常用字段：智能]) AND “注射液”[常用字段：智能] |
|  | Study Design | “随机对照试验”[常用字段：智能] OR “RCT”[常用字段：智能] |

Note:The Chinese database utilizes a Chinese search format.
