# Supplementary material for: The effects of Salvia miltiorrhiza and ligustrazine injection combined with ACEI/ARB on diabetic kidney disease: A systematic review and meta-analysis
Source: Medicine (Baltimore). 2024 Feb 23;103(8):e35853. doi: 10.1097/MD.0000000000035853 (PMC11309681; doi:10.1097/MD.0000000000035853)
Supplement: Supplementary file 2 [file medi-103-e35853-s002.docx]

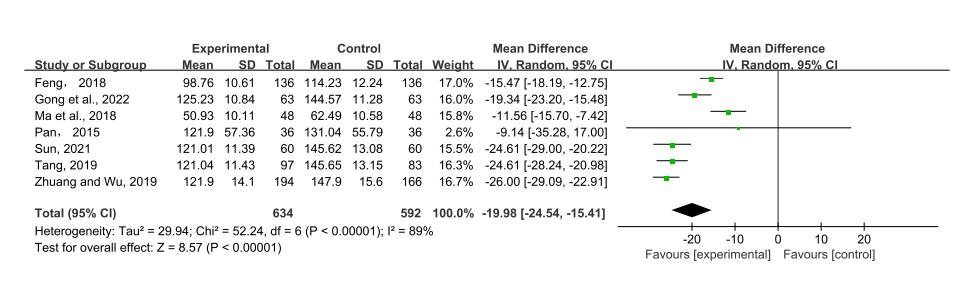


**Fig. 1-1.** Effects of SML combined with ACEI vs. ACEI on Scr.


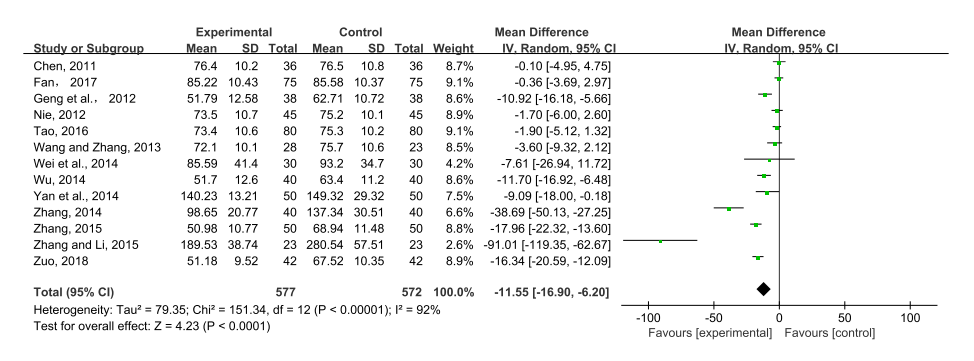


**Fig. 1-2.** Effects of SML combined with ARB vs. ARB on Scr.


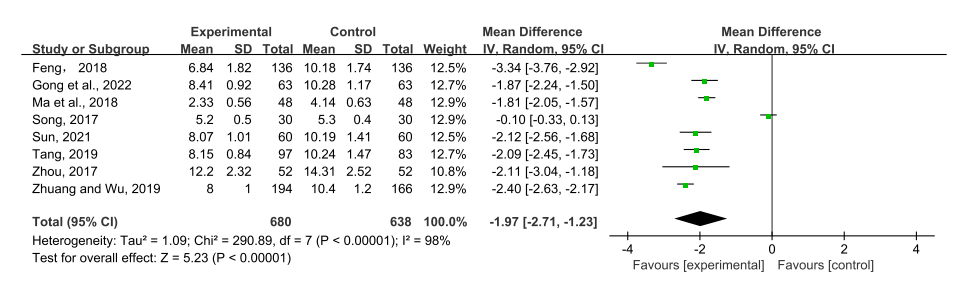


**Fig. 1-3.** Effects of SML combined with ACEI vs. ACEI on BUN.


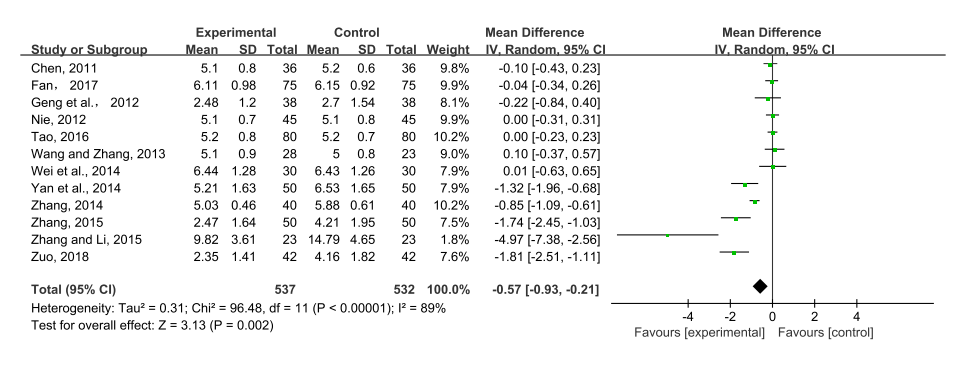


**Fig. 1-4.** Effects of SML combined with ARB vs. ARB on BUN.


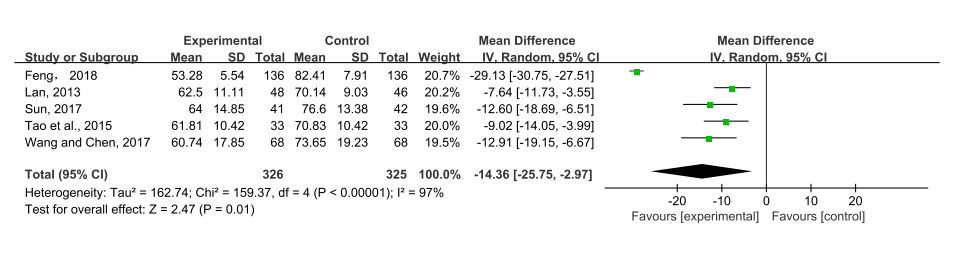


**Fig. 1-5.** Effects of SML combined with ACEI vs. ACEI on UAER.


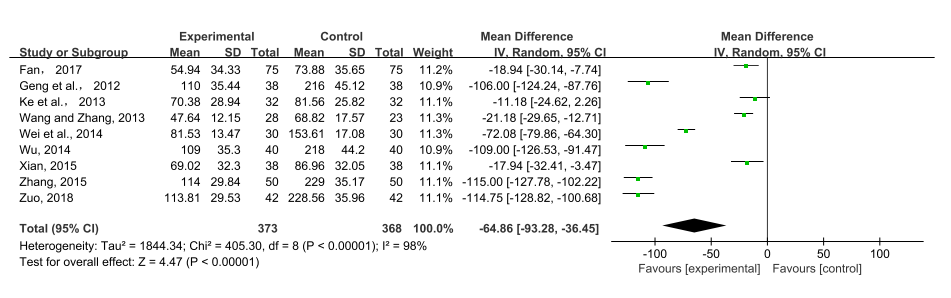


**Fig. 1-6.** Effects of SML combined with ARB vs. ARB on UAER.


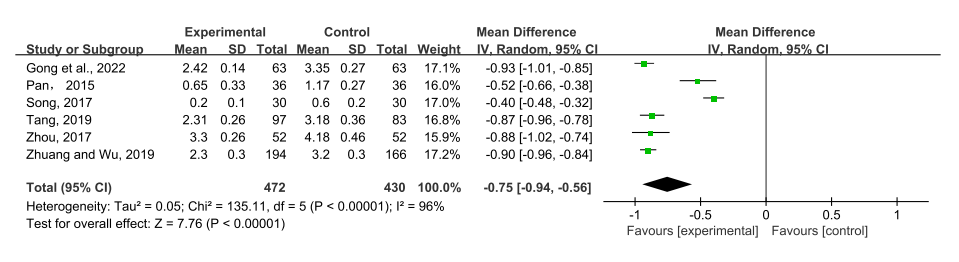


**Fig. 1-7.** Effects of SML combined with ACEI vs. ACEI on 24-UTP.


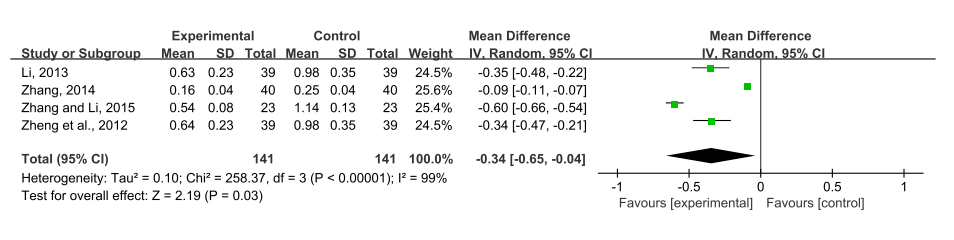


**Fig. 1-8.** Effects of SML combined with ARB vs. ARB on 24-UTP.
