# Supplementary material for: The effects of Salvia miltiorrhiza and ligustrazine injection combined with ACEI/ARB on diabetic kidney disease: A systematic review and meta-analysis
Source: Medicine (Baltimore). 2024 Feb 23;103(8):e35853. doi: 10.1097/MD.0000000000035853 (PMC11309681; doi:10.1097/MD.0000000000035853)
Supplement: Supplementary file 3 [file medi-103-e35853-s003.docx]

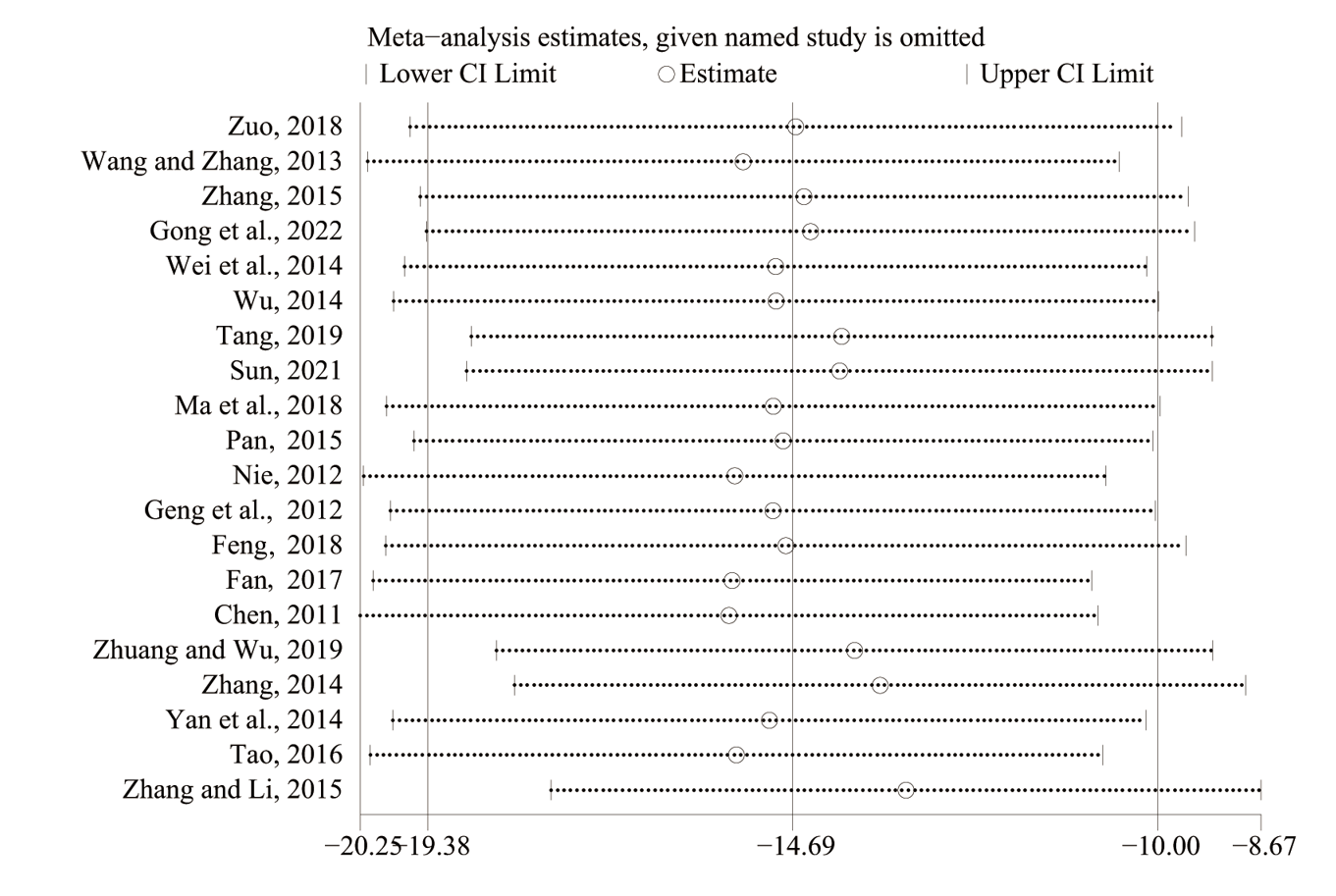


**Fig. 2-1.** Sensitivity Analysis of SCr.


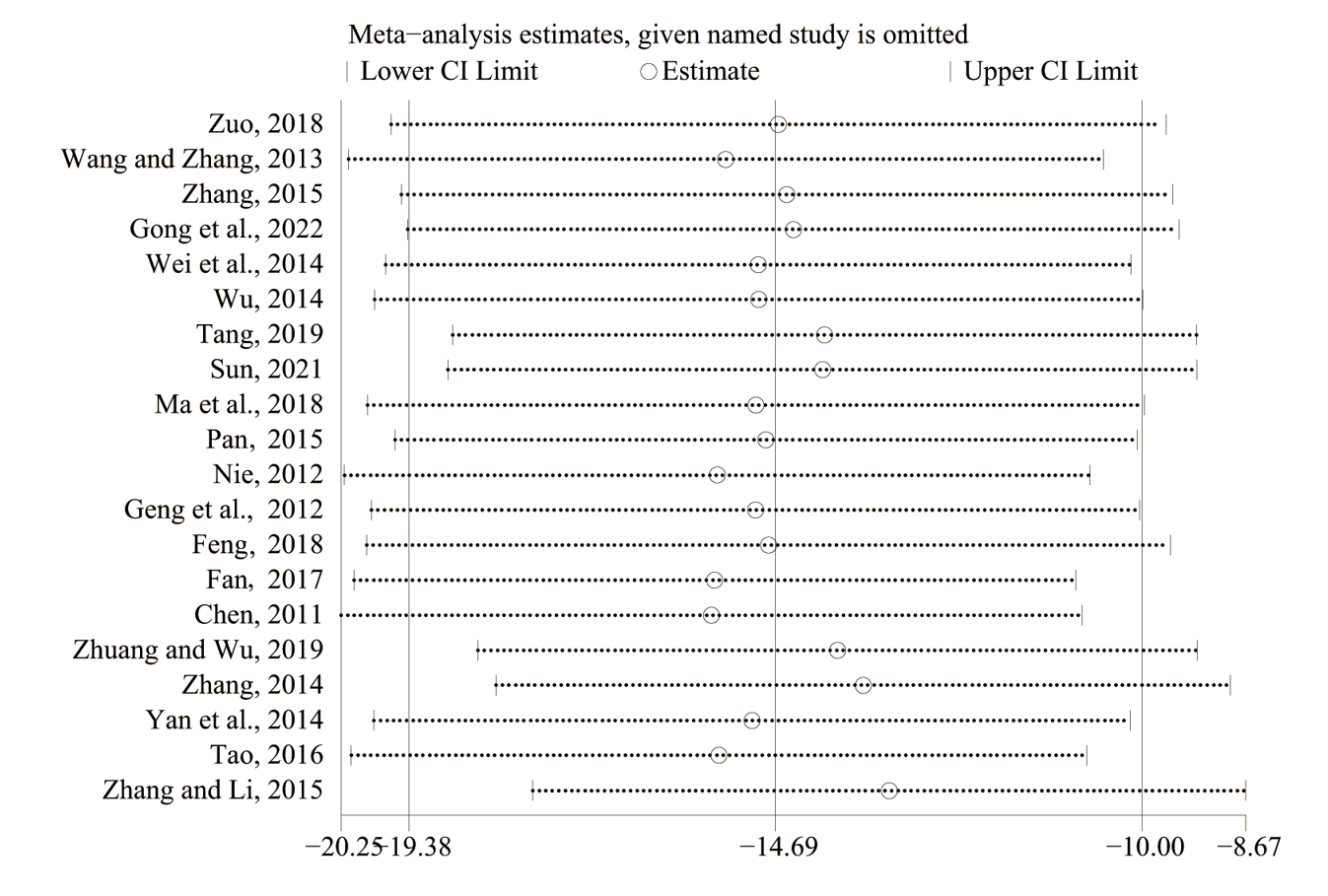


**Fig. 2-2.** Sensitivity Analysis of BUN.


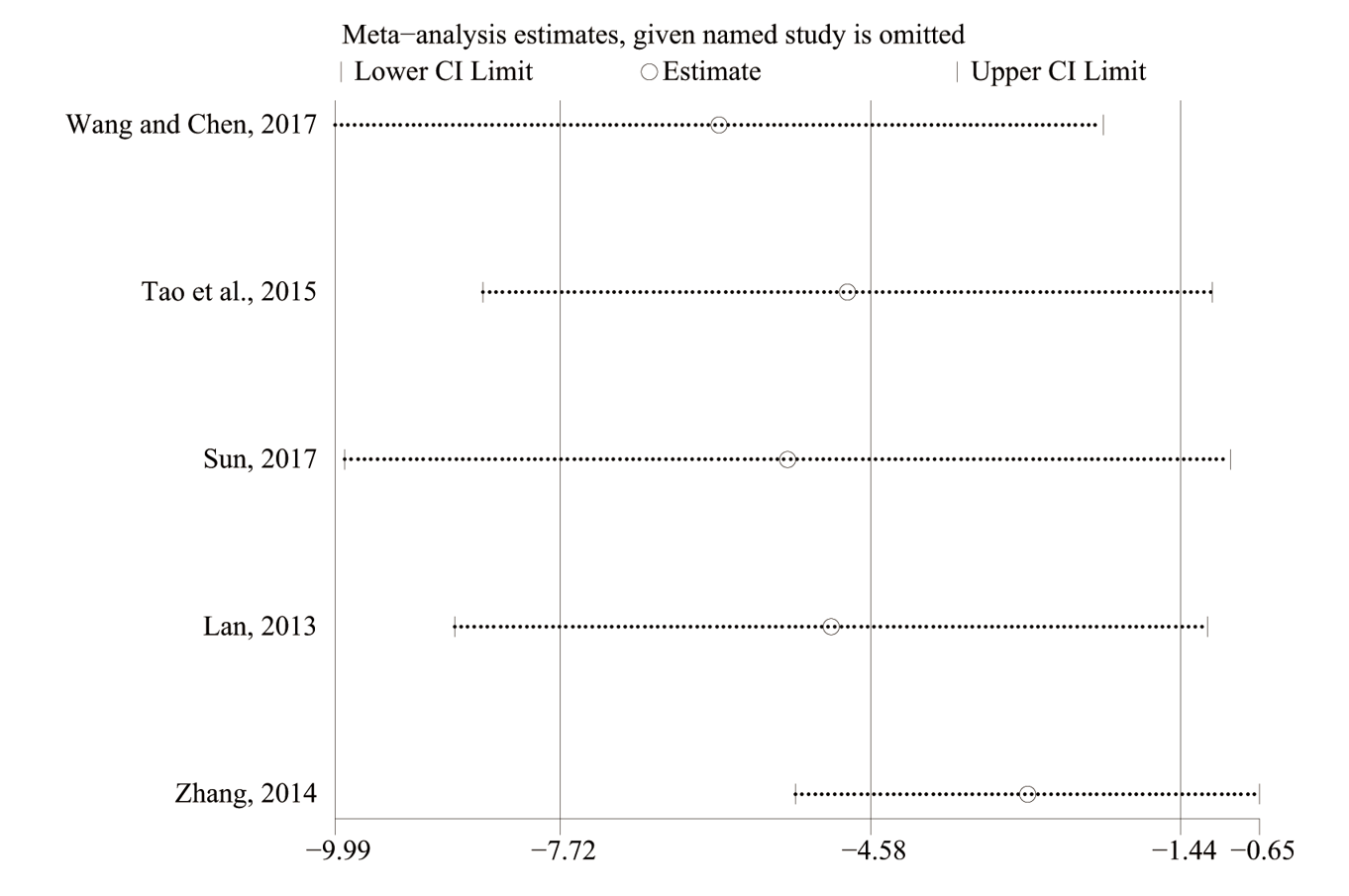


**Fig. 2-3.** Sensitivity Analysis of β2-MG.


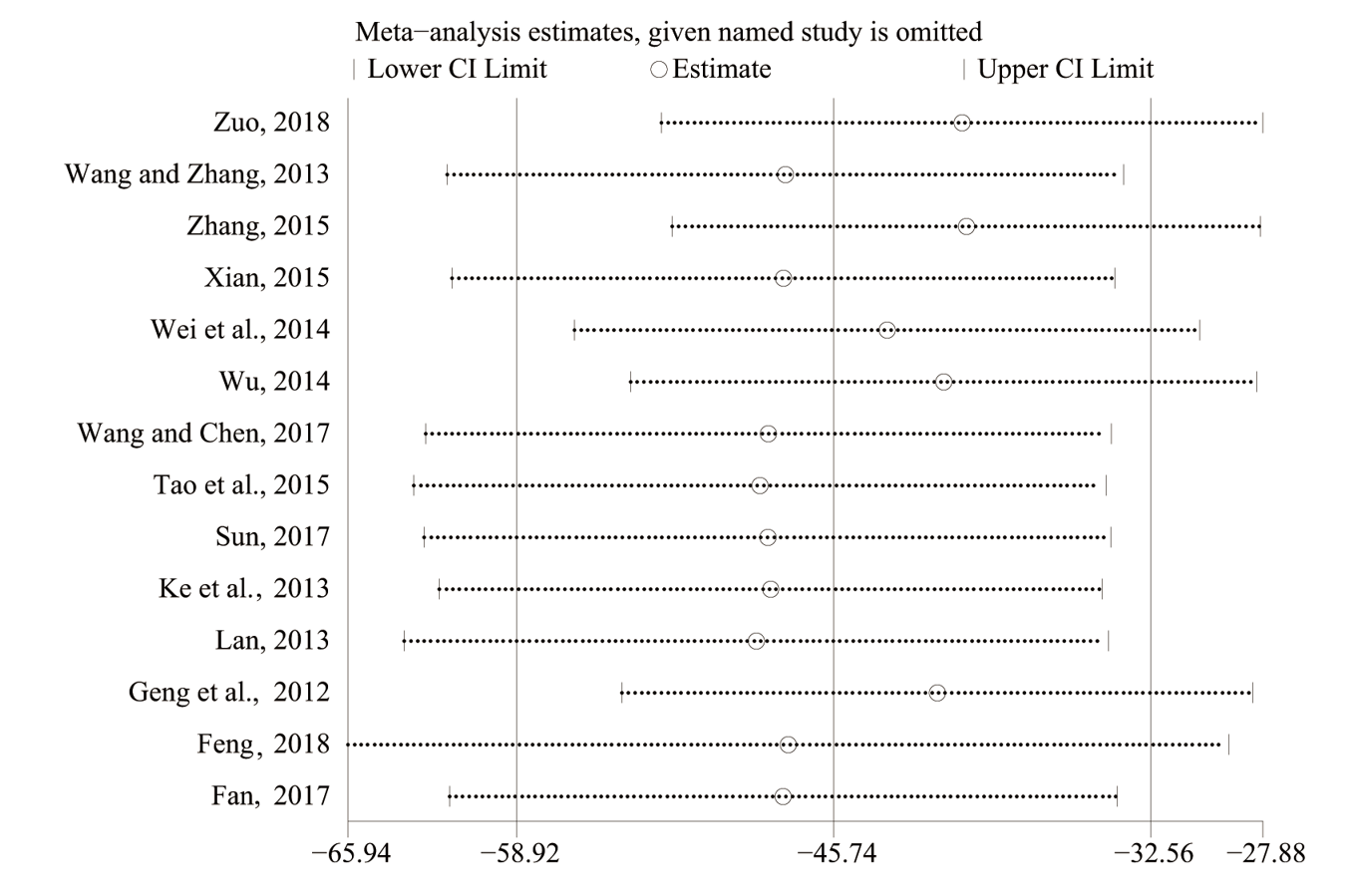


**Fig. 2-4.** Sensitivity Analysis of UAER.


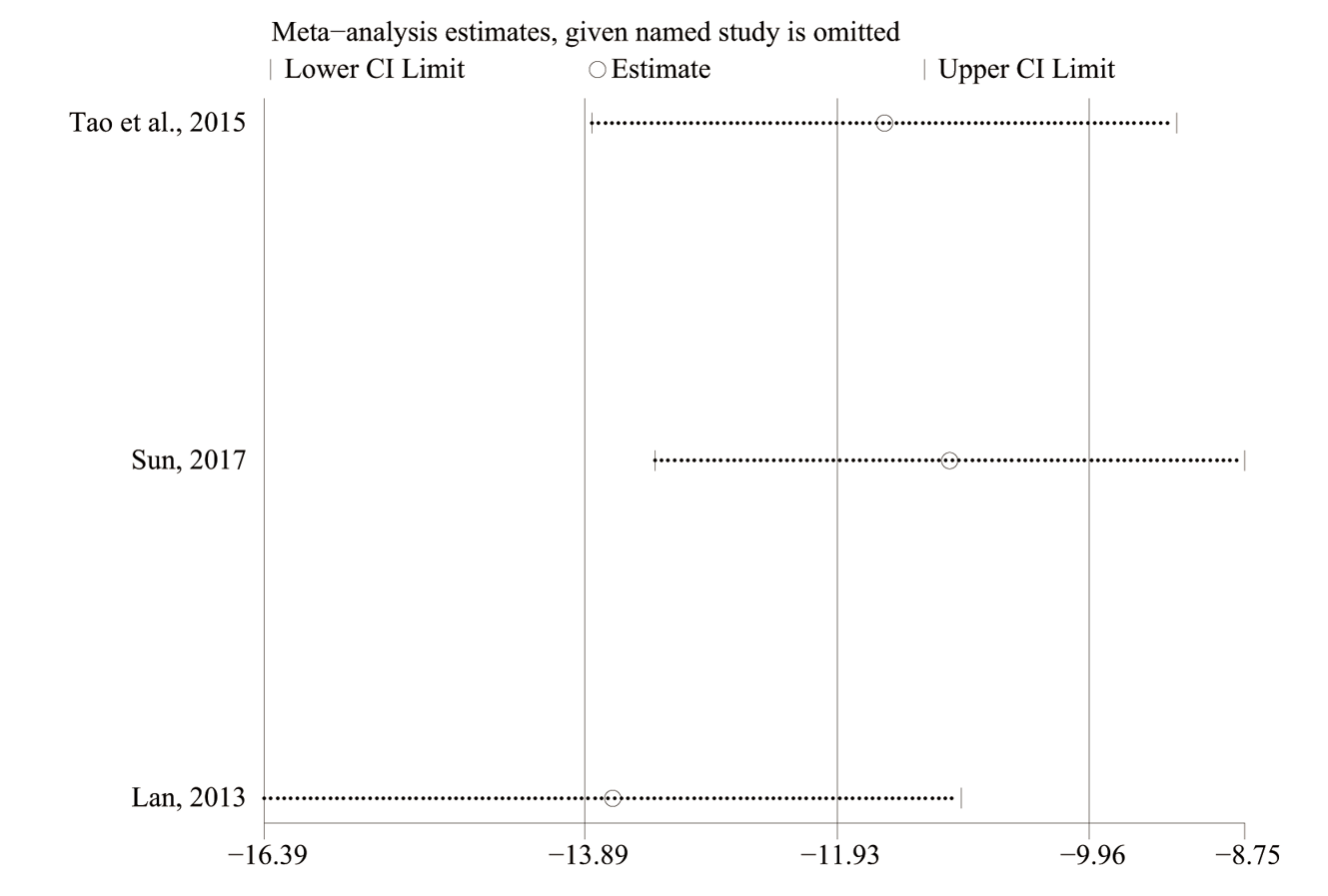


**Fig. 2-5.** Sensitivity Analysis of UACR.


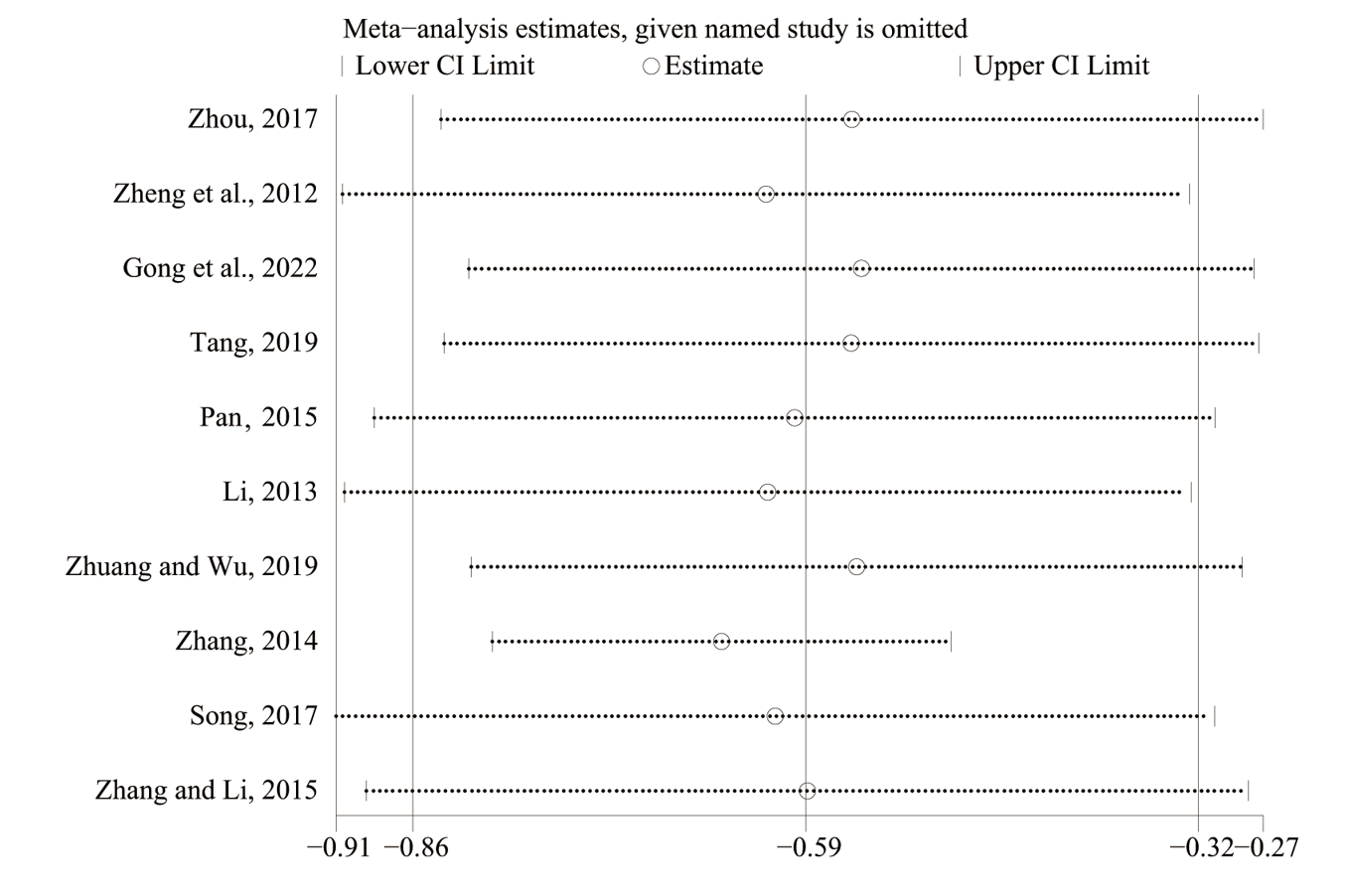


**Fig. 2-6.** Sensitivity Analysis of 24h-UTP.


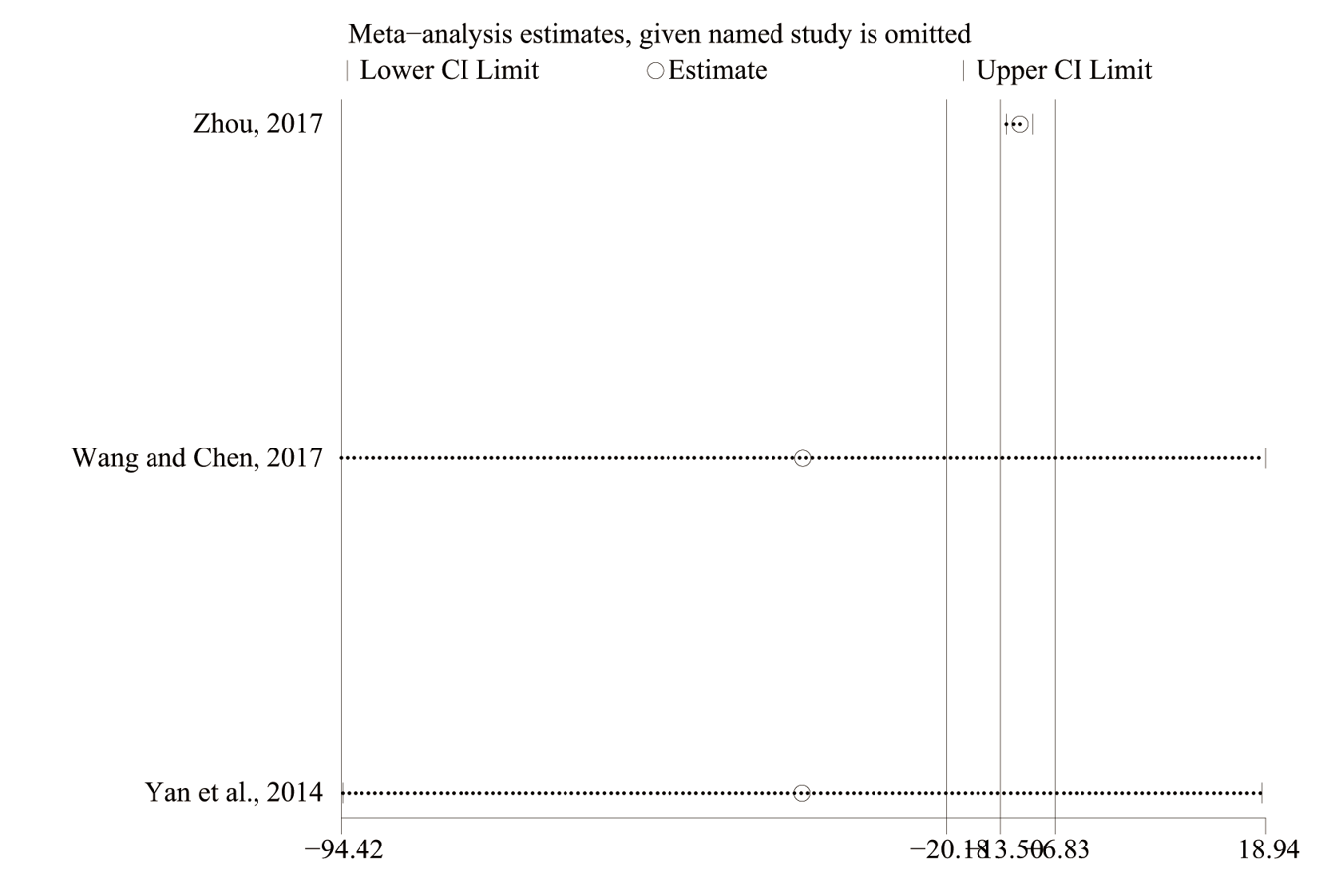


**Fig. 2-7.** Sensitivity Analysis of mALB.


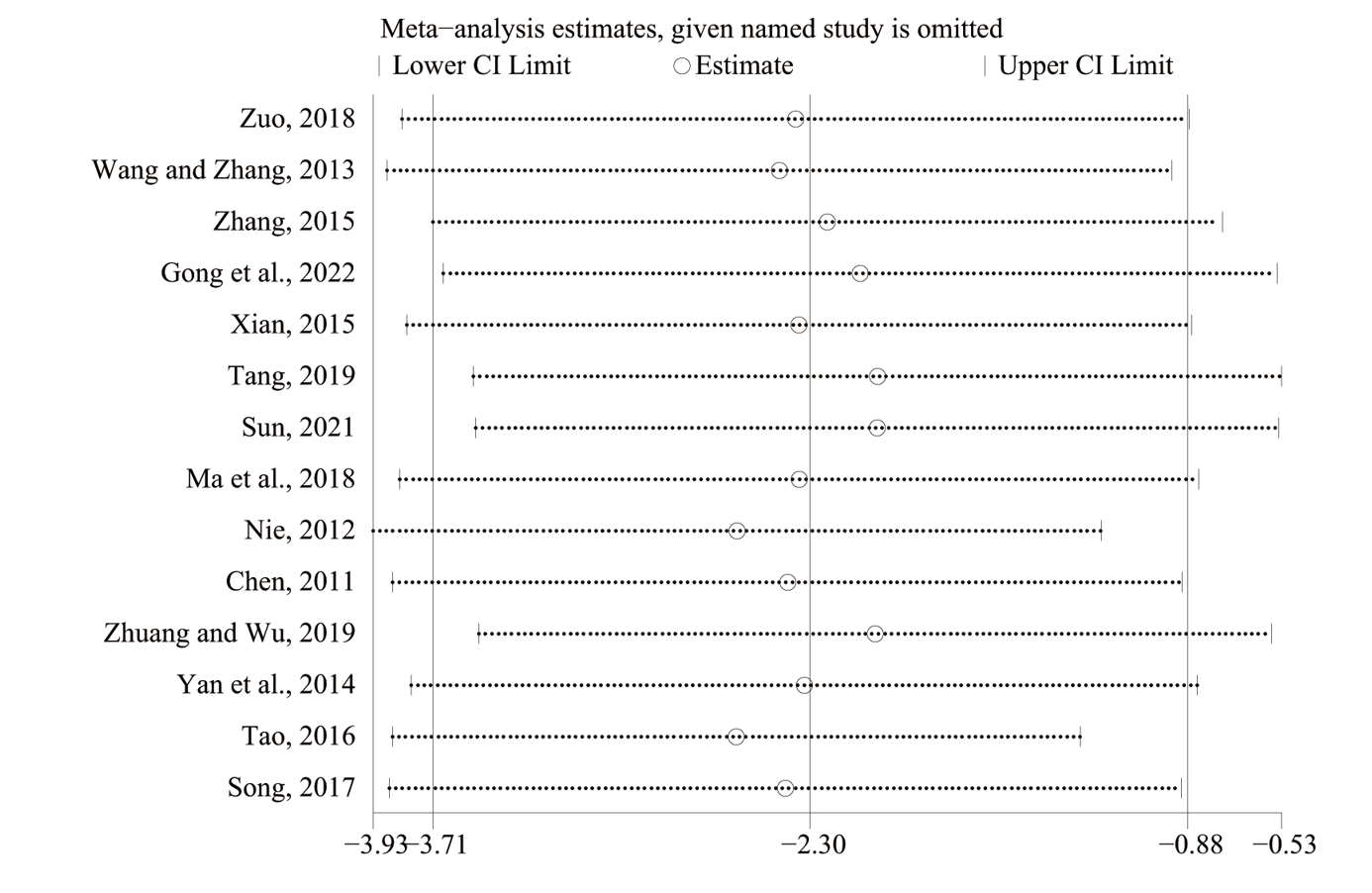


**Fig. 2-8.** Sensitivity Analysis of 2hPBG.


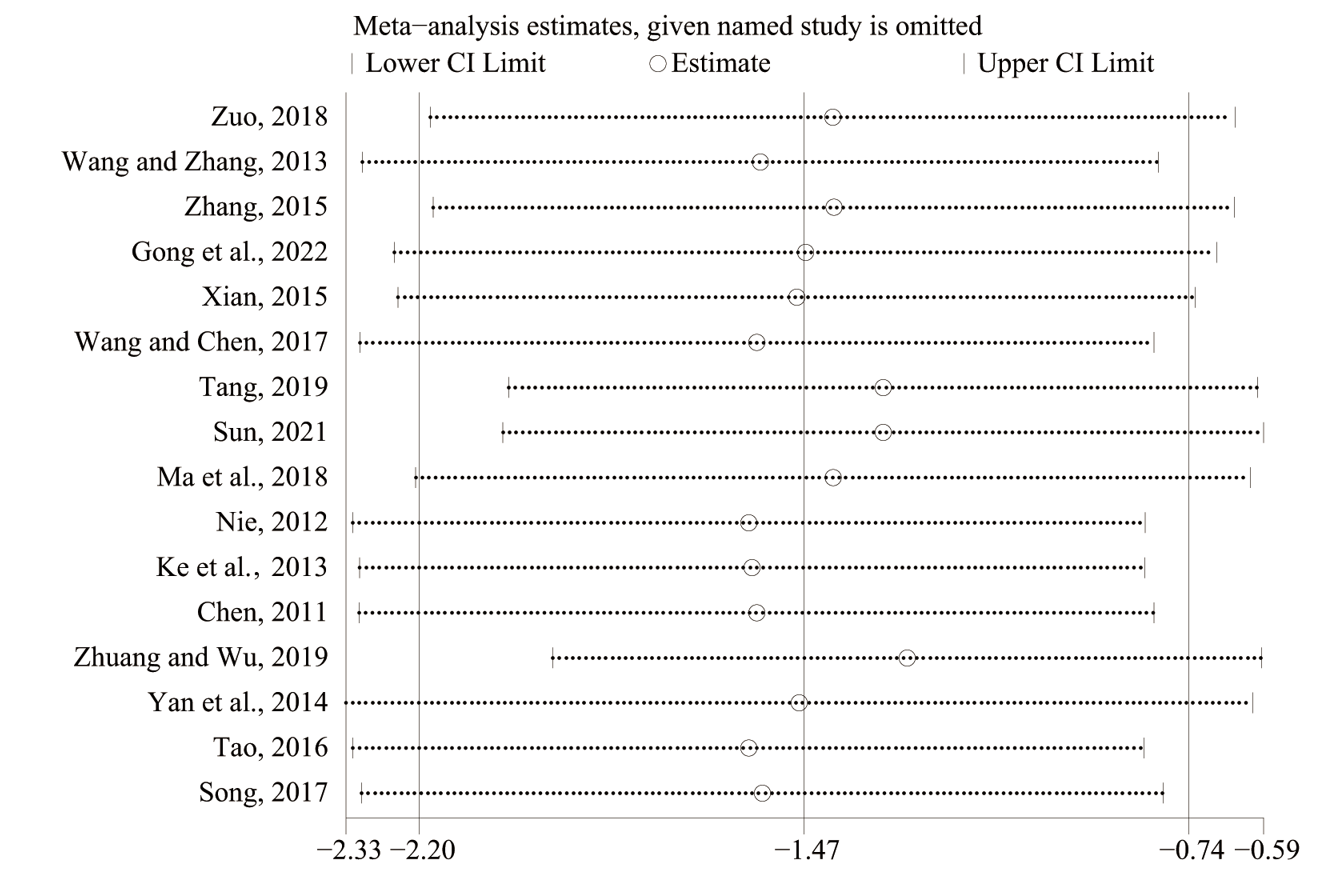


**Fig. 2-9.** Sensitivity Analysis of FPG.


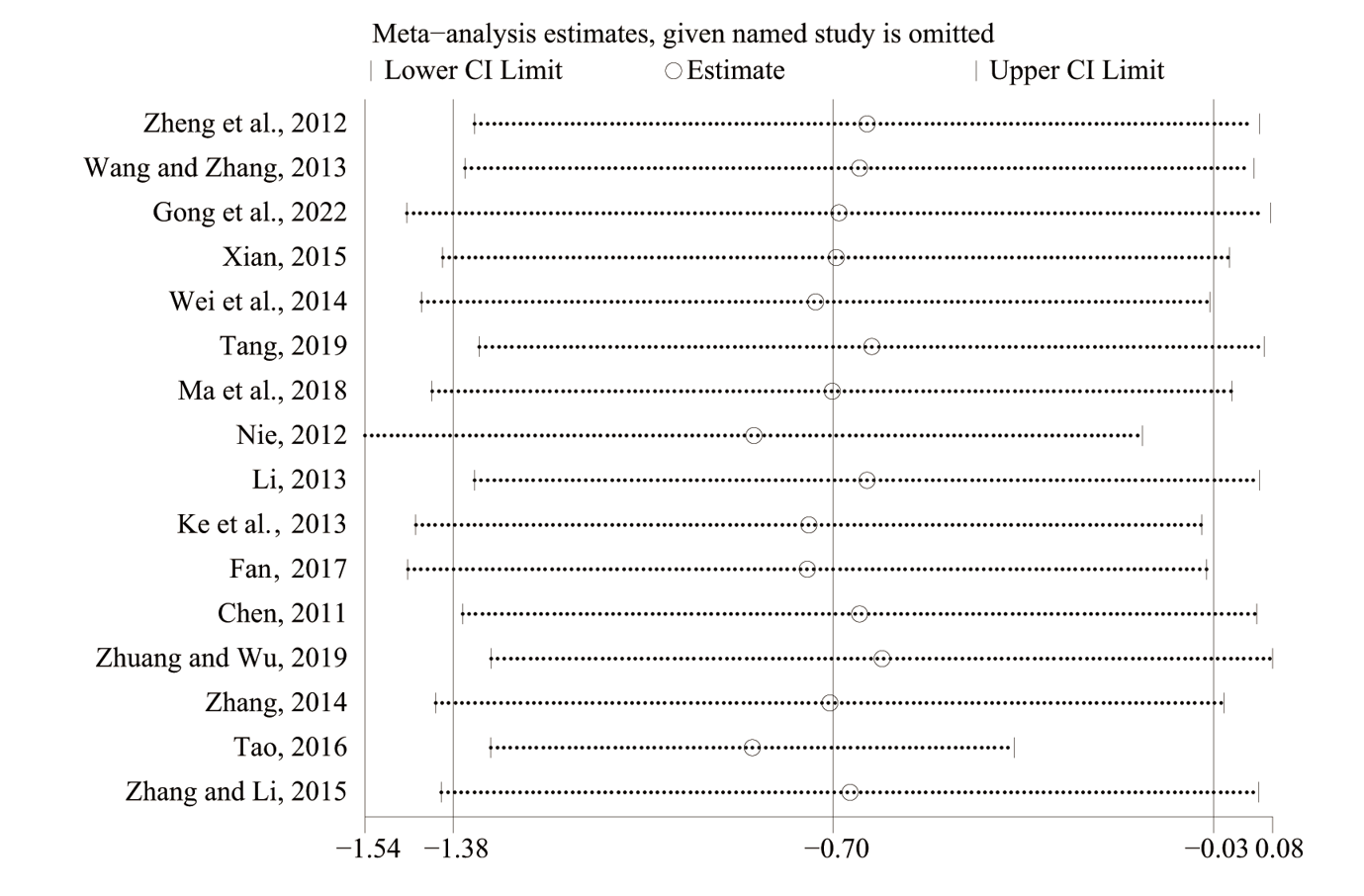


**Fig. 2-10.** Sensitivity Analysis of HbA1c.


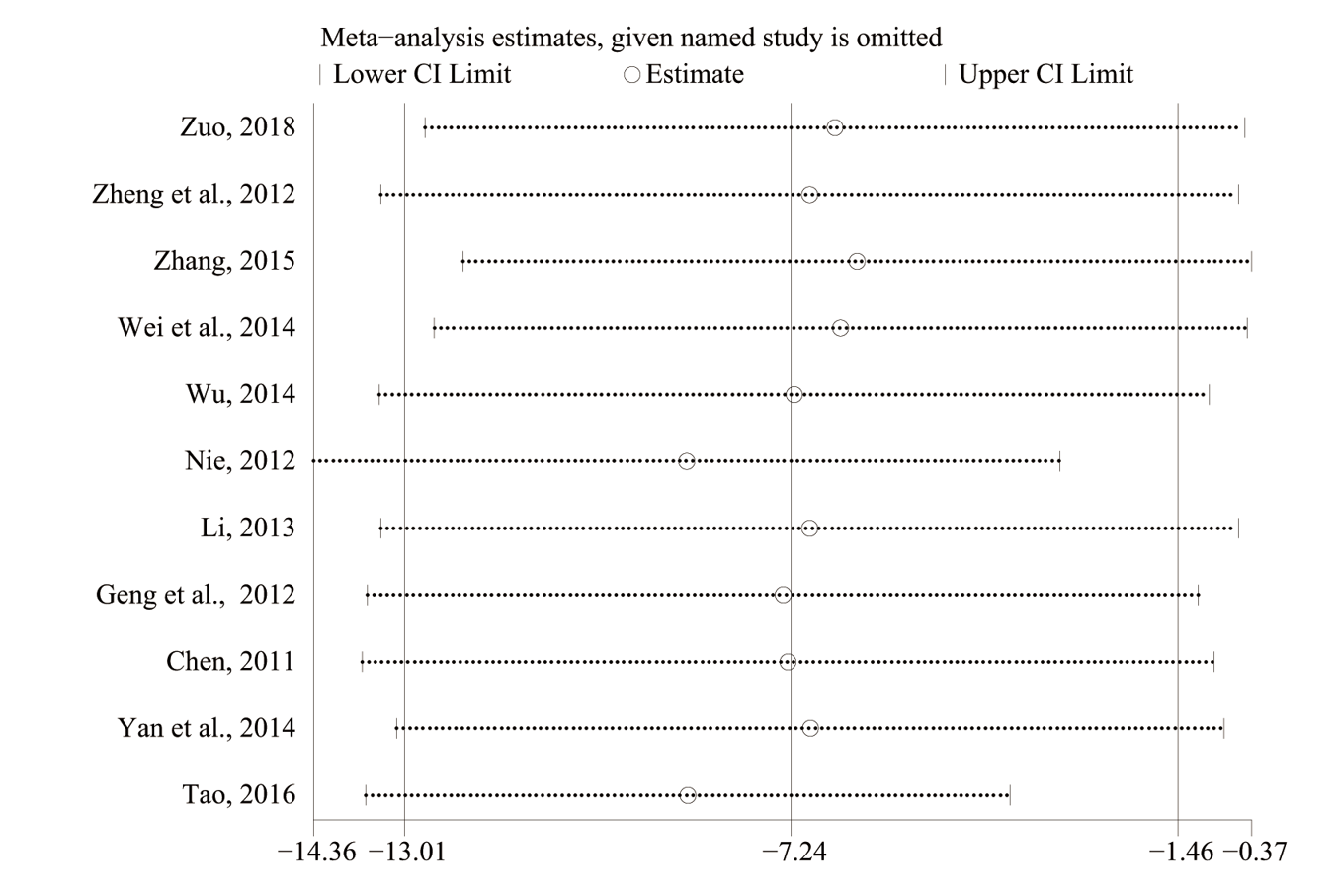


**Fig. 2-11.** Sensitivity Analysis of SBP.


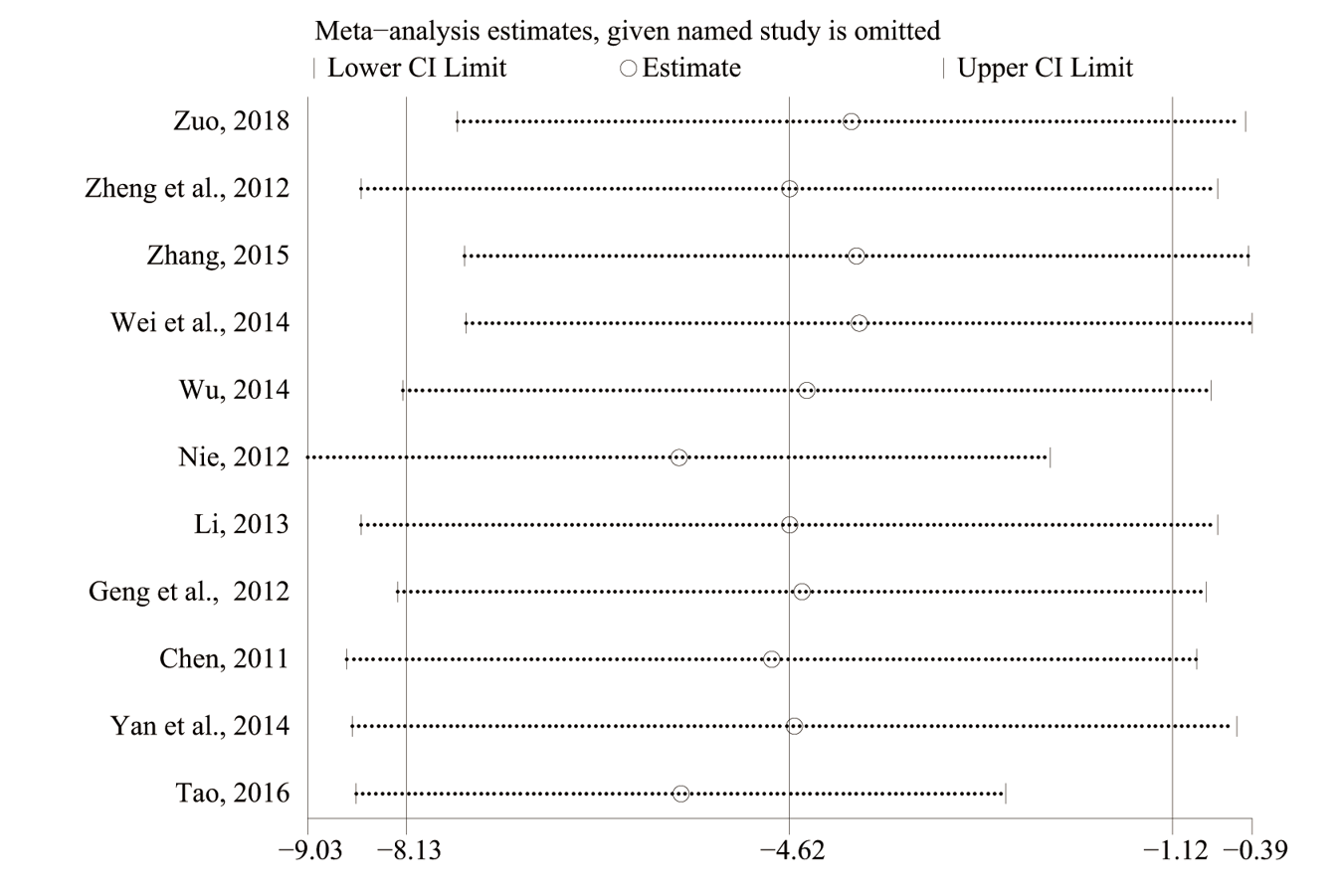


**Fig. 2-12.** Sensitivity Analysis of DBP.


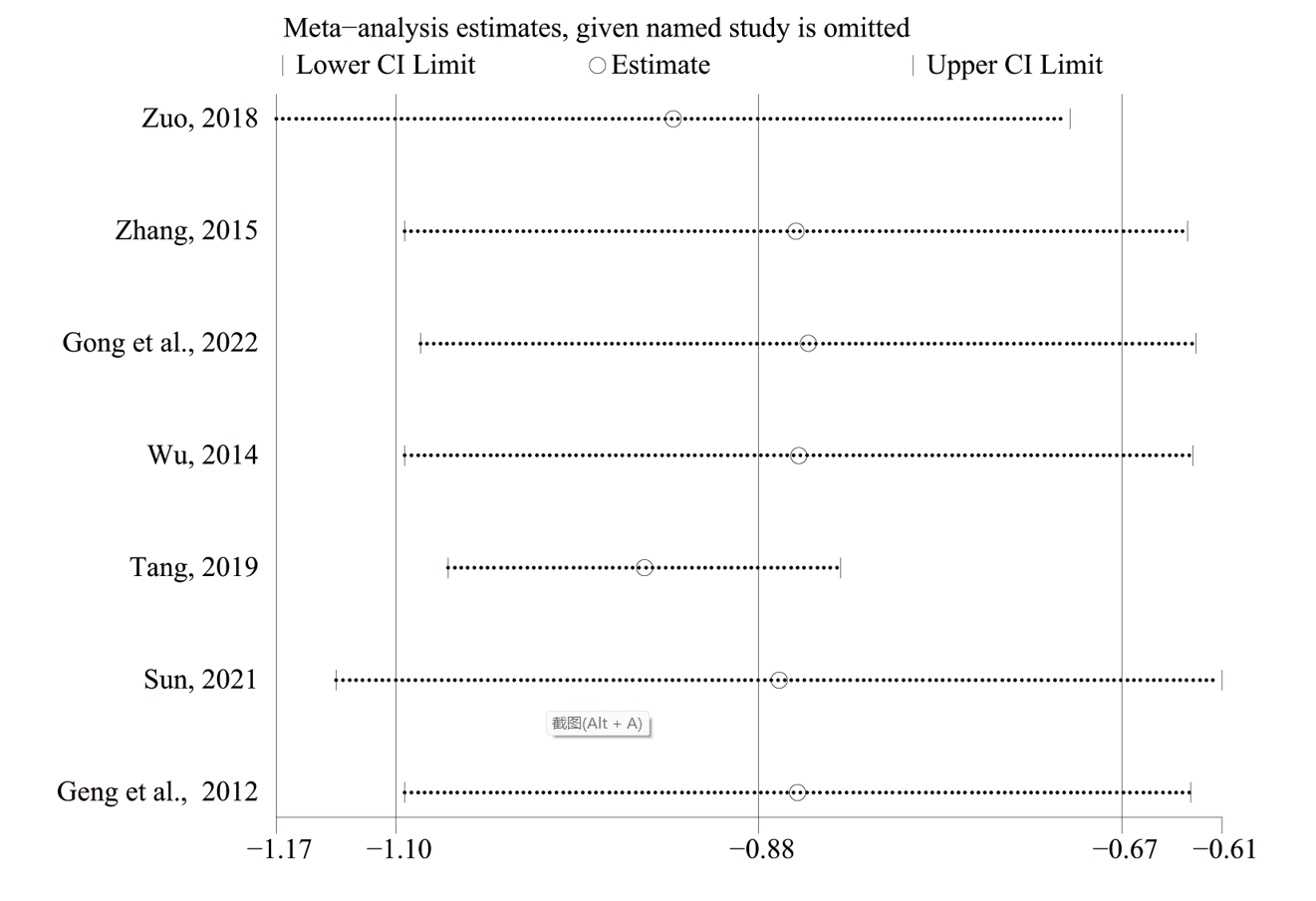


**Fig. 2-13.** Sensitivity Analysis of TG.


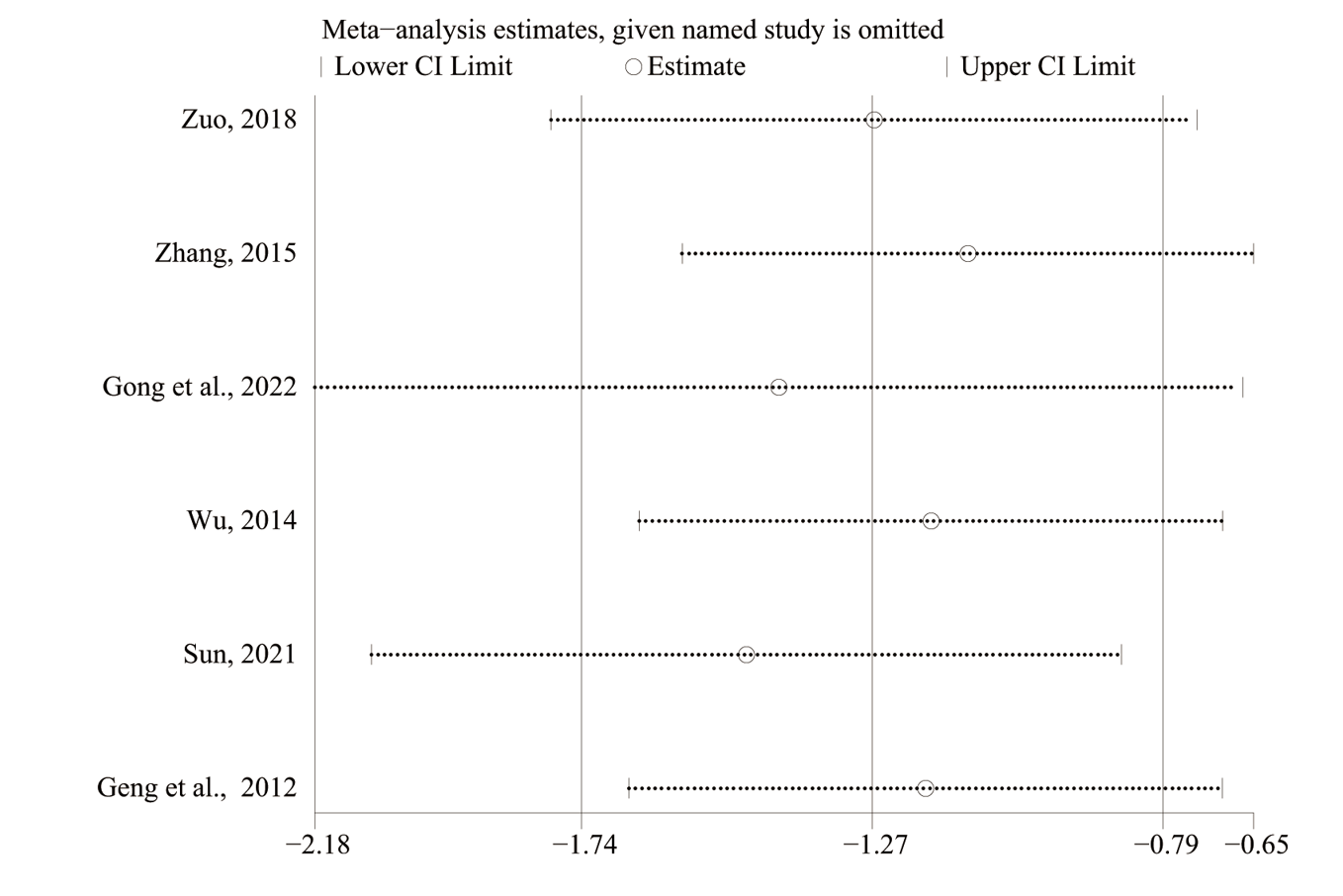


**Fig. 2-14.** Sensitivity Analysis of TC.


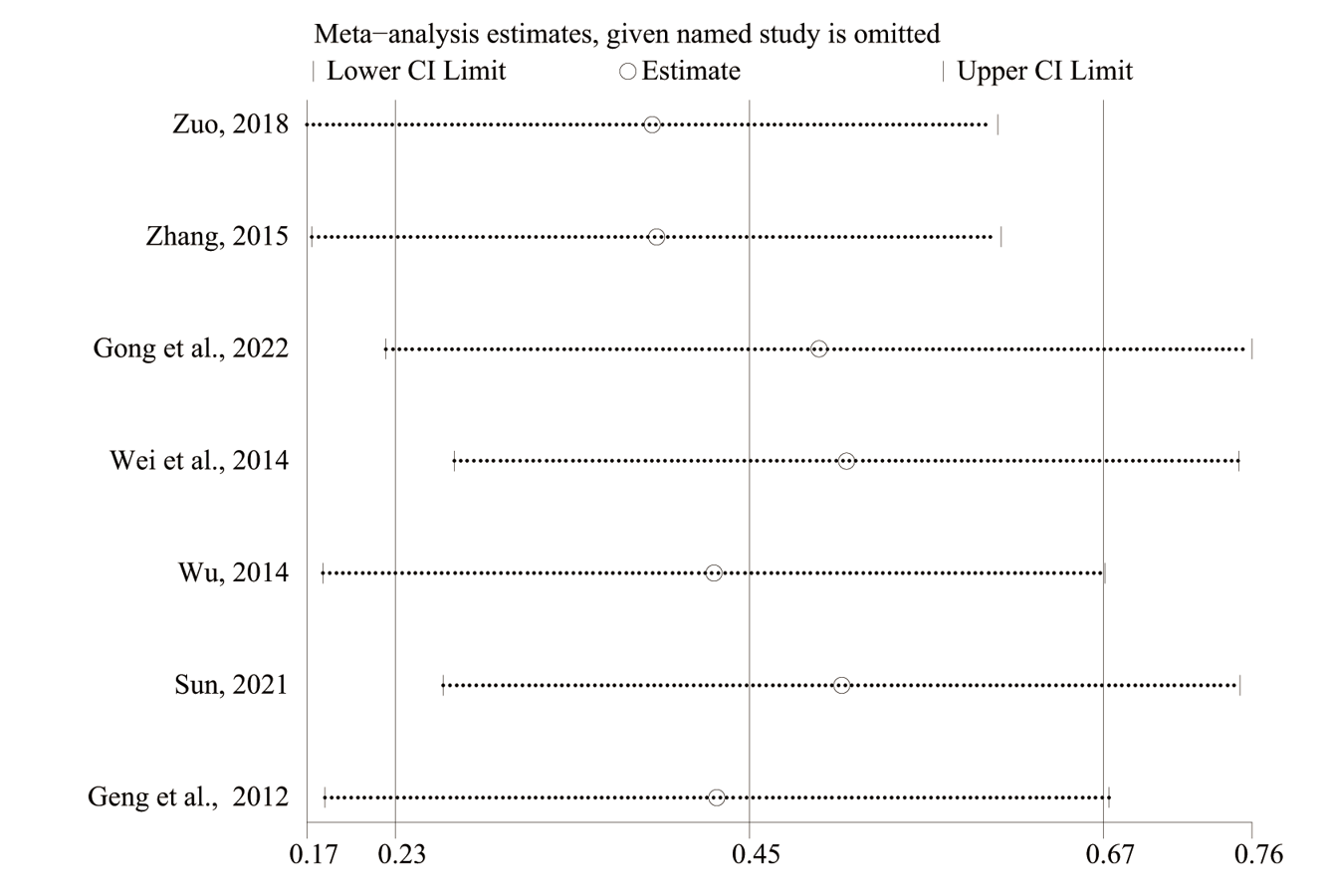


**Fig. 2-15.** Sensitivity Analysis of HDL-C.


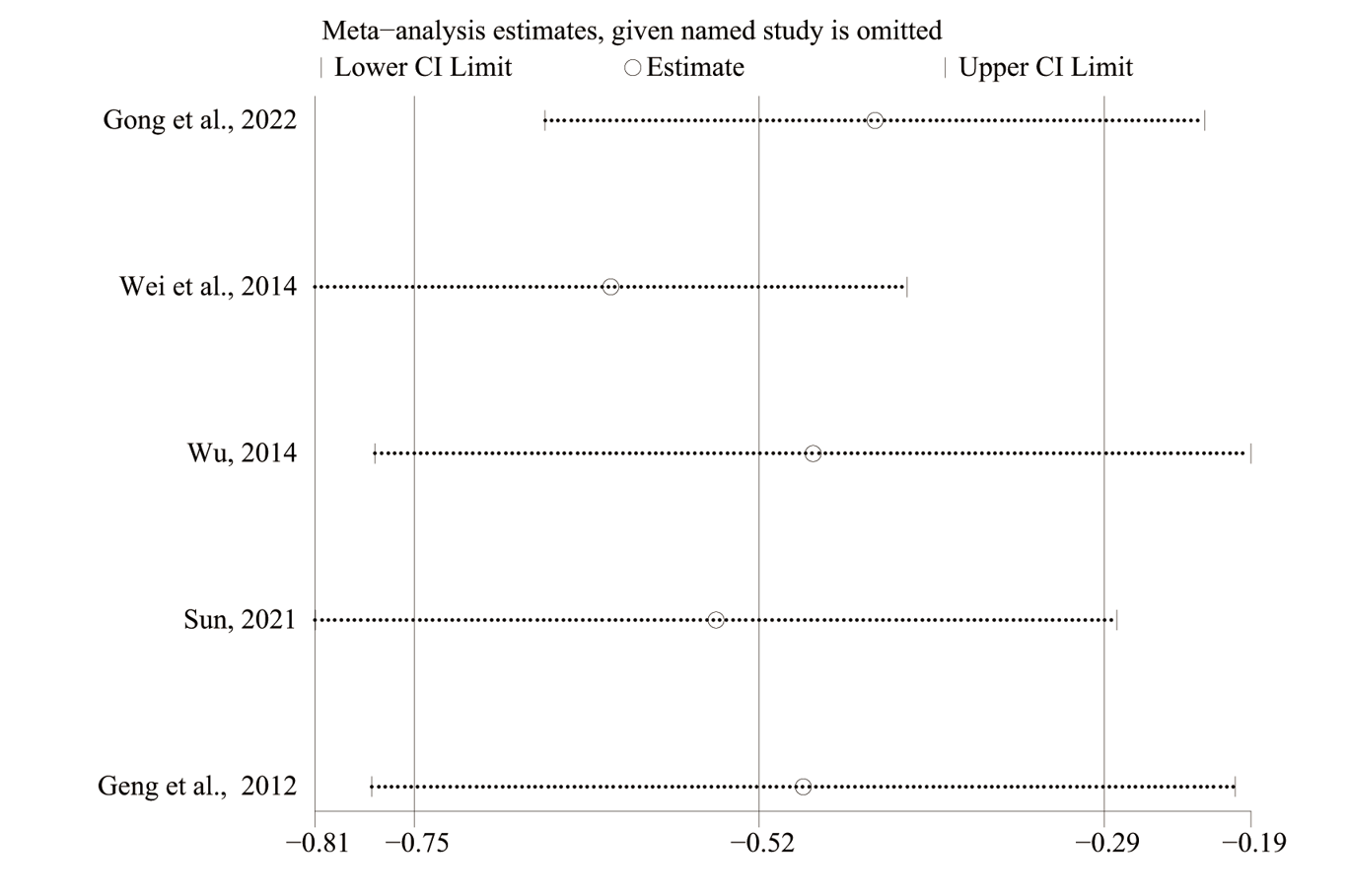


**Fig. 2-16.** Sensitivity Analysis of LDL-C.


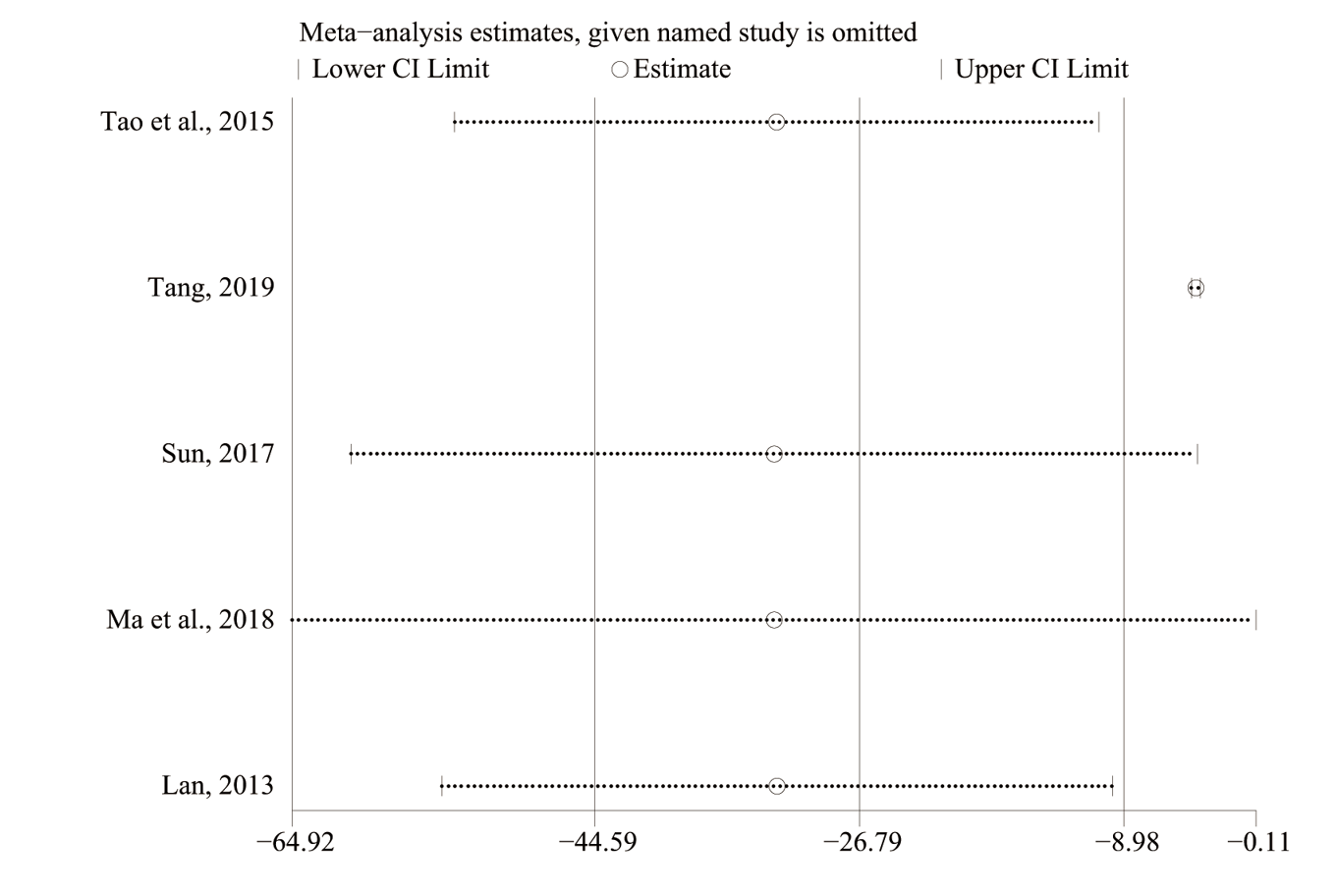


**Fig. 2-17.** Sensitivity Analysis of IL-6.


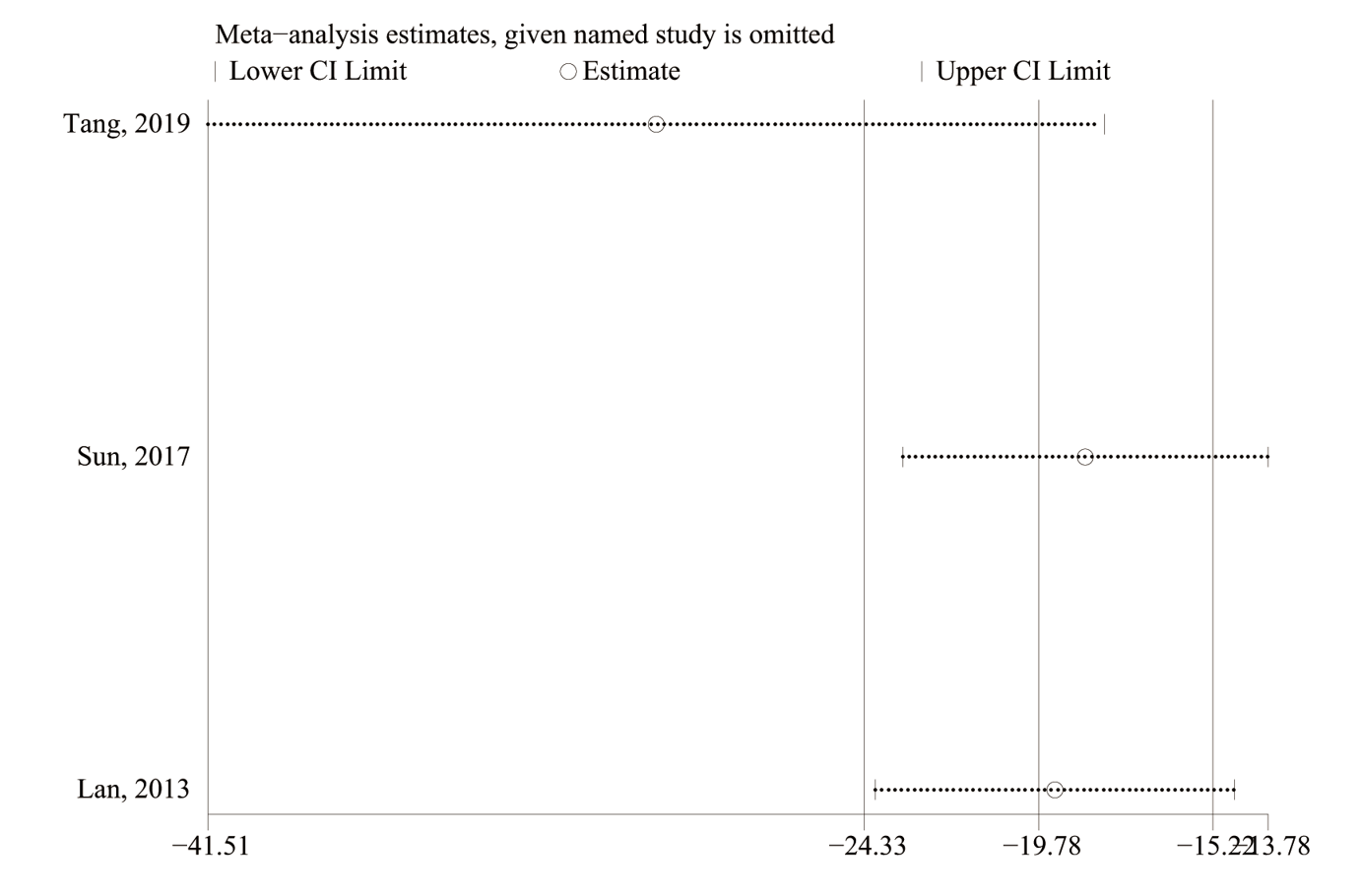


**Fig. 2-18.** Sensitivity Analysis of IL-18.


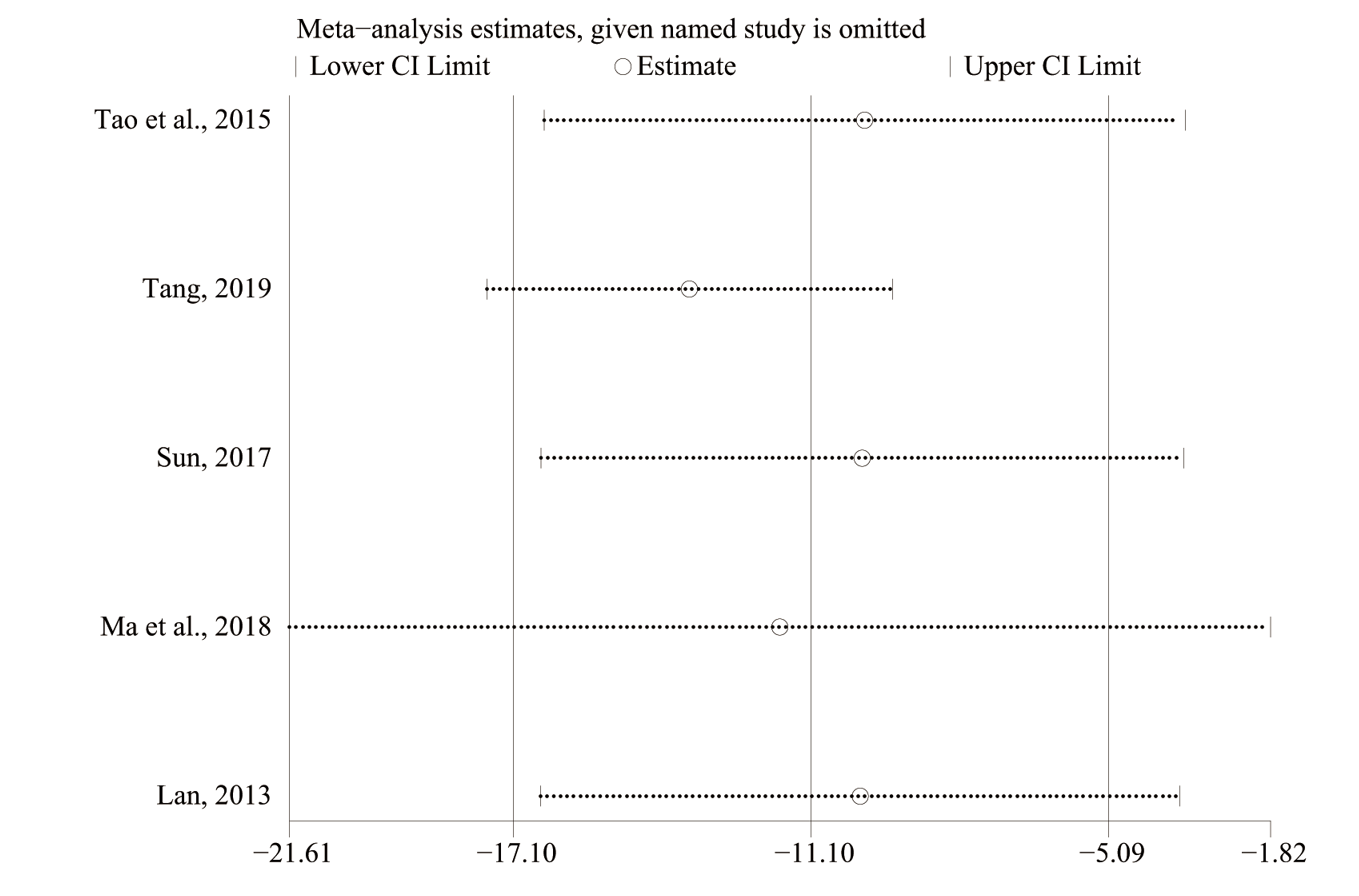


**Fig. 2-19.** Sensitivity Analysis of TNF-α.
