# Supplementary figures and images for: The effects of Salvia miltiorrhiza and ligustrazine injection combined with ACEI/ARB on diabetic kidney disease: A systematic review and meta-analysis
Source: Medicine (Baltimore). 2024 Feb 23;103(8):e35853. doi: 10.1097/MD.0000000000035853 (PMC11309681; doi:10.1097/MD.0000000000035853)

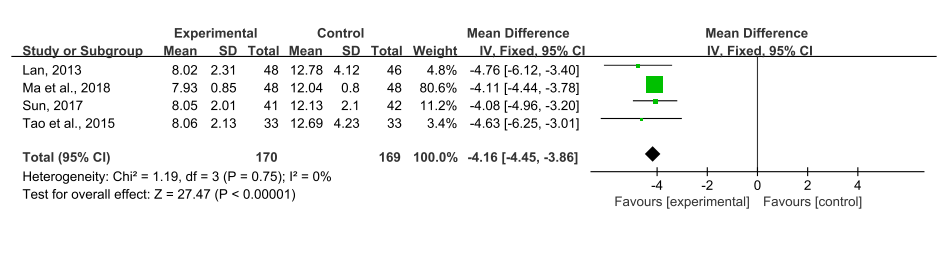


**Fig. 3-1.** The forest plot of IL-6.


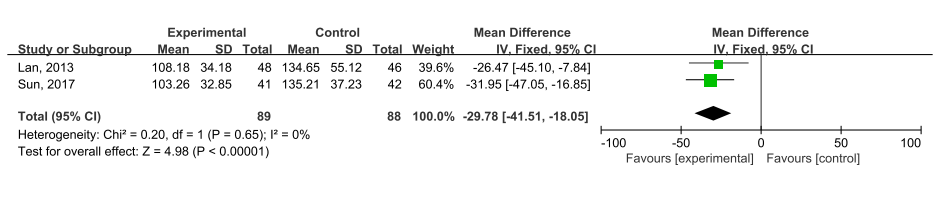


**Fig. 3-2.** The forest plot of IL-18.


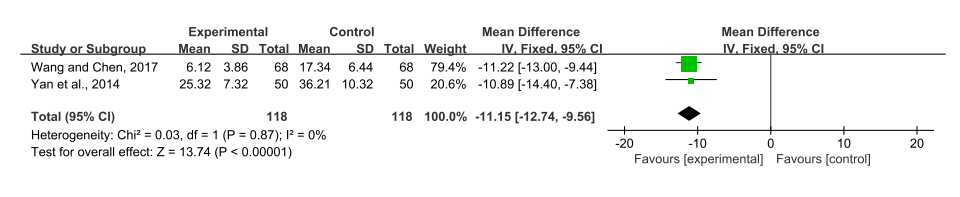


**Fig. 3-3.** The forest plot of mALB.

Supplement: Supplementary file 4 [file medi-103-e35853-s004.docx]
